# Establishment of bladder cancer spheroids and cultured in microfluidic platform for predicting drug response

**DOI:** 10.1002/btm2.10624

**Published:** 2023-12-04

**Authors:** Qiao Xiong, Ting Liu, Yidie Ying, Xiaowen Yu, Ziwei Wang, Hongliang Gao, Tianhai Lin, Weihua Fan, Zhensheng Zhang, Qiang Wei, Yuqing Ge, Shuxiong Zeng, Chuanliang Xu

**Affiliations:** ^1^ Department of Urology Institute of Urology, West China Hospital, Sichuan University Chengdu P. R. China; ^2^ Department of Urology Changhai Hospital, Naval Medical University Shanghai P. R. China; ^3^ State Key Laboratory of Transducer Technology Shanghai Institute of Microsystem and Information Technology, Chinese Academy of Sciences Shanghai P. R. China; ^4^ Department of Geriatrics Changhai Hospital, Naval Medical University Shanghai P. R. China

**Keywords:** bladder cancer, chemotherapy sensitivity, microfluidic chip, personalized treatment, tumor spheroid

## Abstract

Cisplatin‐containing combination chemotherapy has been used as the standard treatment for bladder cancer patients at advanced stage. However, nearly 50% of patients are nonresponders. To guide the selection of more effective chemotherapeutic agents, a bladder cancer spheroids microfluidic drug sensitivity analysis system was established in this study. Bladder cancer spheroids were established and successfully cultured in a customized microfluidic device to assess their response to different chemotherapeutic agents. The in vitro drug sensitivity results were also compared to patient‐derived xenograft (PDX) models and clinical responses of patients. As a result, bladder cancer spheroids faithfully recapitulate the histopathological and genetic features of their corresponding parental tumors. Furthermore, the in vitro drug sensitivity outcomes of spheroids (*n* = 8) demonstrated a high level of correlation with the PDX (*n* = 2) and clinical response in patients (*n* = 2). Our study highlights the potential of combining bladder cancer spheroids and microfluidic devices as an efficient and accurate platform for personalized selection of chemotherapeutic agents.

**Translational Impact Statement**

Bladder cancer is a heterogeneous disease as distinct responses to first‐line chemotherapy were observed, with a high resistance rate to chemotherapy. The establishment of tumor models for drug screening is essential. In this study, we cultured patient‐derived tumor spheroids in microfluidic chips for drug sensitivity tests. It was proven that drug responses of spheroids closely resembled patients undergoing chemotherapy. The clinical translation of this study may help identifying nonresponders before chemotherapy and optimize the adjuvant therapy of bladder cancer patients.

## 1 INTRODUCTION

Bladder cancer is one of the most common urinary tract neoplasms; the worldwide age‐standardized incidence rate is 9.5 and 2.4 per 100,000 person/years for men and women, respectively. The standard treatment for patients with muscle‐invasive bladder cancer is radical cystectomy, but it only provides 5‐year survival rate of about 50%.^1^ Cisplatin‐containing combination chemotherapy has been used as the standard treatment for advanced‐stage bladder cancer since the 1980s, with an overall survival of 12–14 months reported in different studies.^2^, ^3^ However, more than 50% of patients with urothelial carcinoma (UC) are not eligible for cisplatin‐based chemotherapy, and a significant portion of eligible patients (46%–49%) are nonresponders.^4^ Recently, immune checkpoint inhibitors such as pembrolizumab or atezolizumab are recommended as standard treatment for patients who are ineligible for or unresponsive to platinum‐based chemotherapy.^1^ Therefore, identifying non‐responders and providing alternative checkpoint inhibitors to these individuals is crucial in the clinical management of bladder cancer, as it can help reduce chemotherapy‐related toxicities and costs. Currently, there are still no tools available in clinical practice to select patients who are more likely to benefit from cisplatin‐based chemotherapy.^1^

To provide effective clinical solution for selecting sensitive chemotherapeutic agent and achieving individualized treatment, drug sensitivity tests in vitro or in vivo prior to clinical treatment would be of significant value. The most commonly used in vitro models for studying biological processes and drug sensitivity screening in bladder cancer are cell lines, they are easy to use, consistent, and reproducible.^5^ However, cell lines fail to recapitulate crucial biological features and tumor microenvironment of a growing tumor in vivo, making them less clinically relevant for individual patients.^6^ Although patient‐derived xenografts (PDXs) have been developed as another important preclinical model, this method is time‐consuming, expensive, labor‐intensive, and not suitable for high‐throughput drug screening.^7^, ^8^ Recently, patient‐derived organoid lines of UC were established for tumor evolution and drug response studies. Organoid lines often retain parental tumor heterogeneity and can recapitulate the drug response observed in PDX models.^9^, ^10^ It was noted that organoid lines are composed only of cancer cells, lacking an immune system and stromal components, they also exhibit phenotypic instability, which are the major limitations of organoids.^11^, ^12^ Although organoid coculture systems exist to better simulate human disease, they are complex, and lack a standardized protocol or guidelines.^13^

The tumor spheroids have been established to mimic the cellular heterogeneity of the solid tumors, as well as the drug resistance mediated by the tumor‐stromal cells.^14^ In the present study, we successfully cultured spheroids from patient‐derived tumor tissues in vitro for a long period, and demonstrated that these spheroids faithfully recapitulate the histopathological and genetic features of their corresponding parental tumors. Furthermore, we developed a microfluidic device to observe how spheroids responded to the first‐line chemotherapy regimen for bladder cancer and proved the utility of these patient‐derived spheroids as an effective model for drug sensitivity assay.

## 2 MATERIALS AND METHODS

### 2.1 Clinical samples

We obtained tumor specimens and follow‐up data from patients who underwent either transurethral resection of the bladder or radical cystectomy according to protocols approved by the Ethics Committee of Changhai Hospital (No. CHEC2017‐124). Informed consent was obtained from all participants. The results of drug sensitivity assays were blinded to the patients and were not intended to be used for the selection of chemotherapy regimens in patient management. Around 0.5 cm^3^, tumor tissue was obtained from each patient and placed in a 15 mL sterile centrifugal tube containing culture medium within 0.5 h after dissection of the tumor. We randomly selected two pieces of tumor tissue and stored at −80°C for DNA isolation, and the rest for spheroids and PDX model preparation. Histological and immunohistochemical (IHC) information of the corresponding tumor sample was obtained from the Department of Pathology, Changhai Hospital.

### 2.2 Culture of spheroids

Figure S1A illustrates the steps involved in the preparation and formation of cancer spheroids from bladder cancer specimens. The tissues were first placed in a sterile 10 cm petri dish on ice and washed by submersion in 1X Hank's balanced salt solution without Ca2+ and Mg2+ (HBSS, Sigma Aldrich) to remove the blood clot. The tissues were further minced into <2 mm pieces and dissociated using collagenase type I (Gibco) diluted in serum‐free 1640 medium (Gibco) with final concentration of 2 μg/mL. Enzymatic digestion was carried out in a 37°C bath/shaker (200 rpm) for 20 min. The pellets were spun down at 800 rpm for 2 min, washed once with serum‐free 1640 medium, and resuspended with endothelial cell medium (ECM) (ScienCell). To obtain spheroids of certain sizes, digested tissue fragments were filtered using sterile strainers (Fischer Scientific) of 100, 70, and 40 μm in turn. Tissue fragments larger than 100 μm could be digested for another 15 min to obtain sufficient cell clusters. Isolated clusters were incubated on the ultralow attachment (ULA) six‐well plate, and cell clusters spontaneously formed spheroids. Spheroids were cultured in ECM supplemented with 5% fetal bovine serum, 1% penicillin/streptomycin solution, and 1% endothelial cell growth supplement.

### 2.3 Whole exome sequencing

The sequencing libraries were prepared using the Agilent High Sensitivity DNA Kit and were sequenced by Illumina NovaSeq with the target sequencing coverage depth at 150X. Raw sequenced reads were quality controlled and cleaned using fastp with the default parameter. All samples passed quality control with a sufficient read depth and a Q30 rate higher than 90%. After alignment with bwa‐mem, the Picard tools in GTAK4 were applied for duplicate marking and base quality score recalibration. Single nucleotide variations (SNVs), short insertion/deletions (InDels), and copy number variation (CNV) were called with GATK toolkit v4.3.0.0. The “‐‐f‐score‐beta” was set at 1 in the “FilterMutectCalls” function. “‐‐number‐of‐changepoints‐penalty‐factor” was set at 5 in the “ModelSegments” function. CNV profiles were visualized with integrated genome viewer (IGViewer) while the SNVs/InDels were processed and visualized with maftools.

### 2.4 Microfluidic device design and fabrication

The device was designed to mimic the hydrodynamic shape of the bladder by using AutoCAD software (Autodesk AutoCAD 2019, Autodesk, San Rafael, CA, USA) and consists of a polydimethylsiloxane (PDMS, Dow Corning, USA) microchamber containing four drop‐shaped guiders and 19 triangular markers for spheroid seeding. The top of the PDMS layer included two reservoirs connected to the inlet channel (5.0 mm diameter) and an outlet channel (10.0 mm diameter depth), respectively. COMSOL Multiphysics 5.6 (COMSOL AB, Stockholm, Sweden) was used for the simulation analysis of drug diffusion and setting the dimensional parameters of the chips. The microfluidic device was fabricated by applying standard soft lithography, and the detailed fabrication steps were shown in the supplementary materials. The fabricated devices were sterilized by 30 min UV exposure, and then surface modification was performed by 12 h incubation of ECM to make it more conducive to spheroid culture.

### 2.5 Drug sensitivity assay

A total of 100 uL of spheroids suspension was introduced into the inlet of the microchamber, and negative pressure was applied at the outlet using a syringe vacuum. Following the introduction of desired number of spheroids in the cell culture chamber, the flow was stopped, and inlet and out reservoirs were filled with 250 μL of culture media. The difference in surface tension between inlet and outlet media reservoirs created a very slow back‐and‐forth flow (on the order of microliters per hour) that enhanced diffusion–dominated delivery of nutrients to cells. According to the clinical protocol and drug metabolism curve, gemcitabine and cisplatin (GC) were used to sequentially treat spheroids for 24 h, and wait for another 24 h before viability staining. The viability of the spheroids (three for each treatment group and the control) was determined using the Calcein/PI Cell Viability/Cytotoxicity Assay Kit (Beyotime, Nanjing, China) following the manufacturer's protocols. Negative and positive control groups were cultured with ECM only or supplemented with 10% of paraformaldehyde (DEPC). Viability was calculated according to the equation:$\left(S^{L}-S^{\mathrm{PC}}\right)/\left[\left(S^{L}-S^{\mathrm{PC}}\right)+\left(S^{D}-S^{\mathrm{NC}}\right)\right]\times 100\%$, where L, D, PC, and NC are the live and dead staining of sample, positive control, and negative control luminescence records, respectively.

### 2.6 Patient‐derived bladder cancer xenografts

Since previous studies suggested that hormones may cause differences in bladder cancer, we used 4–5‐week‐old female NOD‐*scid* IL2Rgamma^null^ (NSG) mice (Shanghai Laboratory Animal Center, SLAC, Shanghai, China) to establish PDX models, so as to reduce potential bias caused by gender differences.^15^, ^16^ Clinical cancer specimens (3–5 mm^3^) were implanted subcutaneously into the flanks of mice. Mice were randomized into each group (six mice per group) when tumor volumes reached 100–300 mm^3^, followed by initiation of drug treatment. Cisplatin was given at a dose of 4 mg/kg intraperitoneally at day 2. Gemcitabine was given by intraperitoneal injection with a dose of 150 mg/kg on days 1, 8, and 15. Drugs were given simultaneously in the combination treatment group. The tumor volume was calculated using the formula (length × width^2^) × 0.5. Tumor volumes and body weight were recorded twice weekly. Mice with tumor diameter ≥ 2 cm or with body weight loss >20% were promptly euthanized. Tumor tissue was taken out from mice and embedded into formalin‐fixed paraffin‐embedded (FFPE). The FFPE was cut into 4 μm sections and used for IHC (MX Biotechnologies, Fuzhou, China) and TdT‐mediated dUTP nick end labeling (TUNEL) staining (Vazyme, Nanjing, China). All animal experiments were performed in accordance with the Guide for the Care and Use of Laboratory Animals and were approved by the Bioethics Committee of the Naval Medical University, and all experiments were performed following the relevant guidelines and regulations of the Naval Medical University.

### 2.7 Statistical analysis

Student's *t*‐tests and two‐way analysis of variance (ANOVA) (or mixed model) in GraphPad Prism software were used for statistical analysis of all experiments. Test statistics were corrected for multiple tests. A minimum of three biological replicates were used for each condition, and the standard deviation is presented as error bars. The number of biological duplicates and significance (*p*) value threshold used for each experiment are listed in captions.

## 3 RESULTS

### 3.1 Establishment of bladder cancer spheroids from patients' tumor tissue

**FIGURE 1:**
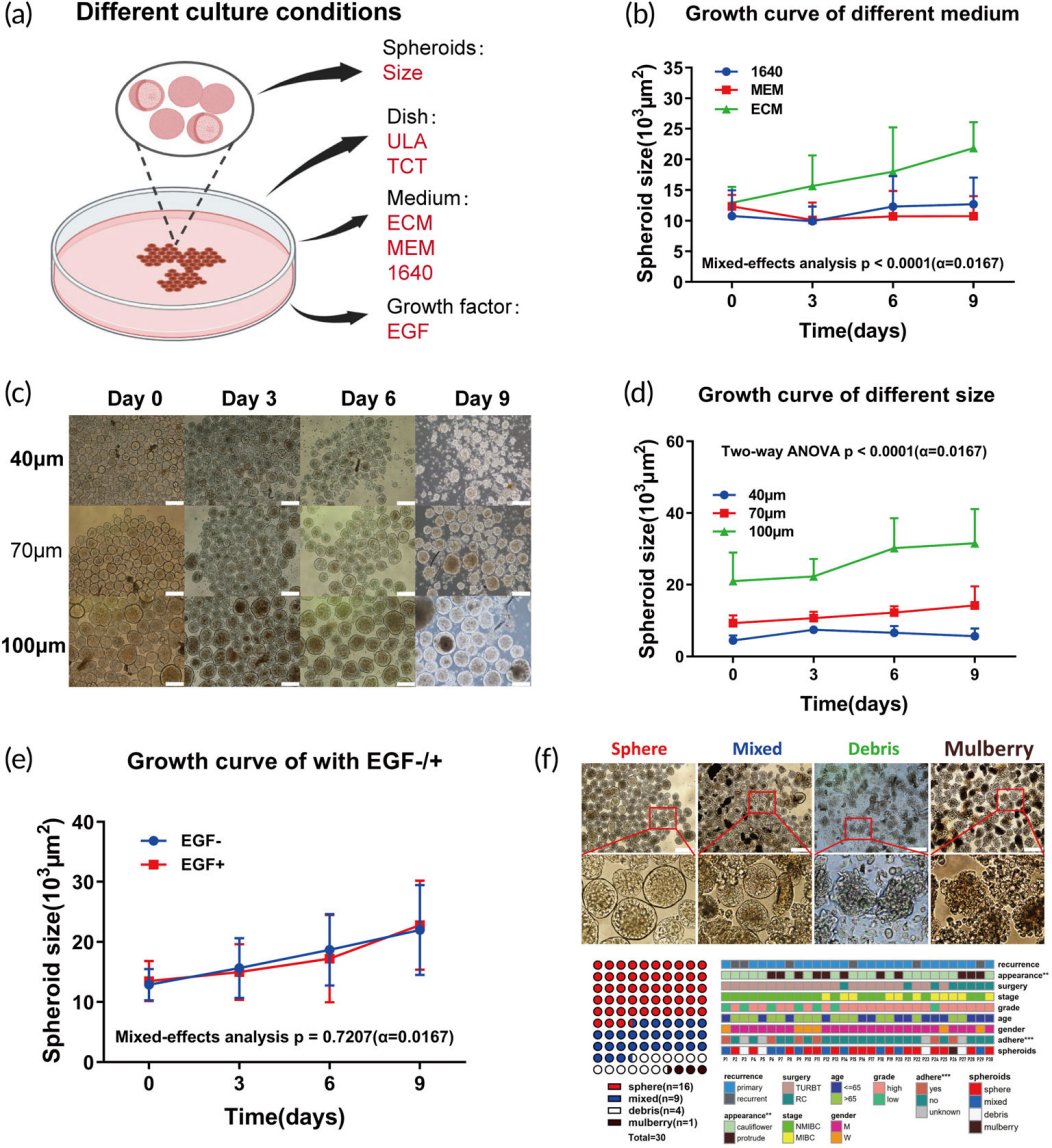
Establishment of patient‐derived bladder tumor spheroids. (a) Schematic of the different conditions we used to culture spheroids in vitro. (b) Tumor spheroids growth curves in different medium culture conditions; *p* < 0.01. (c, d) Representative images of different size spheroids morphology at different time points (c), and spheroids growth curves (d); p < 0.01 (two‐way ANOVA) (scale bar, 200 μm). (e) Tumor spheroids growth curves with or without growth factors. EGF was selected as a representative growth factor to show the effect of additional growth factor on spheroids; *p* = 0.72. (f) Relationship between morphological features of tumor spheroids and clinical; (scale bar, 200 μm); TCT, tissue culture treated surface; ULA, ultralow attachment; EGF, epidermal growth factor.

First, we explored the effect of the different conditions such as medium, spheroids' size, culture dishes, and growth factors on the spheroids culture (Figure 1a). Bladder tumor spheroids tended to grow faster in ECM medium and could maintain the oval shape when cultured in ULA dish (Figures 1b and S1b). After 24 h of culture, we observed that low‐grade tumors tended to be more adhesive and appeared flat on the ULA plates, whereas high‐grade tumors maintained their spheroidal shapes (Figure S1C). Interestingly, as shown in Figure 1c, we observed a different proliferation rate depending on the size of spheroids. Spheroids smaller than 70 μm tended to grow slowly and necrotize, spheroids between 70 and 100 μm, as well as those larger than 100 μm, appeared to have similar growth rates, whereas spheroids larger than 100 μm were prone to developing a necrotic core, possibly due to hypoxia and lack of nutrient (Figures 1d and S1D).^17^ We observed that primary spheroids between 70 and 100 μm in size could maintain their growth rate with or without the addition of extra growth factor (Figure 1e). The overall success rate of the spheroid cultures was 86.7%. It was worth noting that morphological features of tumor spheroids appeared to represent the patient‐specific heterogeneous morphologies, ranging from solid spheroidal to mulberry structures (Figure 1f). We successfully established a total of 30 patient spheroids, and these spheroids were divided into four types according to their morphology (sphere, mixed, debris, and mulberry) (Figures 1f and S1E). We found that spheroid growth beyond two passages may fail due to disruption of tumor microenvironmental cells (e.g., fibroblasts and immune cells). However, spheroids can undergo serial passages through PDXs, ensuring their long‐term viability for further studies (Figure S1F).

### 3.2 Pathological and genetic validation of spheroids

**FIGURE 2:**
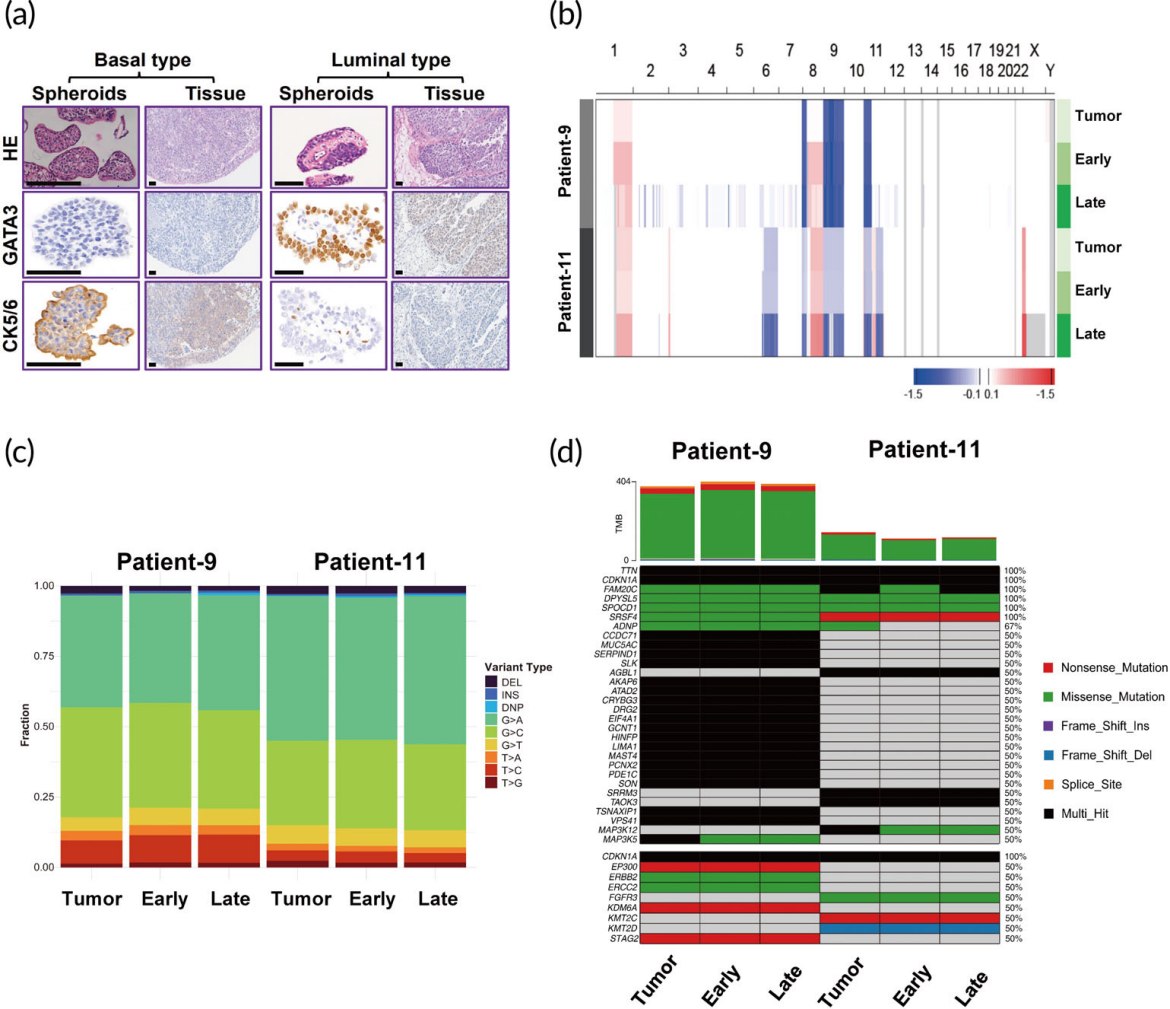
Tumor spheroids maintain histological and genetic features from parental tumor tissue. (a) Representative images of H&E and immunostaining for the indicated basal (CK5/6) and luminal (GATA3) markers. Immunohistochemistry analyses revealed spheroids maintain the distinct subtype of the parental tumor tissue (scale bar, 50 μm). (b) Genome‐wide CNV landscapes identified in spheroids with different culture days in line with their parental tumor. (c) The proportions of exonic somatic variants across the samples from parental tumor (T) and spheroids obtained at day 10 (early) and day 20 (late). Six types of SNV changes, InDels, and DNP are represented. (d) Somatic mutations are shared within the parental tumor and corresponding spheroids (upper). Top 30 most frequently altered genes in this cohort (lower); oncogenes altered in more than 10% of TCGA bladder cancer cohort.

To test whether the bladder cancer spheroids faithfully recapitulate the histological patterns of parental tumor samples, we prepared paraffin sections from spheroids cultures and performed hematoxylin–eosin (H&E) and IHC staining. At the histological level, we demonstrated that the morphology and grading of the spheroids exhibited were consistent with their corresponding parental tumors. Moreover, the IHC expression profile (GATA3, CK5/6) of basal/luminal classification from spheroids resembled that of its corresponding parental tumor (Figure 2a). We then sought to determine whether the spheroids retain the molecular features. Therefore, we performed whole‐exome sequencing analysis of spheroids and the corresponding tumor tissue. At the molecular level, spheroids exhibited multiple chromosomal aberrations, which were in agreement with the corresponding tumor tissue (Figure 2b). The analysis of the proportion of exonic variations confirmed that both single‐nucleotide variants and indels in the original tissue were well retained in spheroids culture. Additionally, the distribution of base substitutions for both tumor tissue and spheroids revealed the most frequently occurring somatic base substitutions were C: G > T: A and C: G > G: C, which aligns with the mutational spectrum described for bladder cancer in previous reports (Figure 2c).^18^, ^19^ Furthermore, we found tumor tissues and matched spheroids had similar tumor mutation burdens and oncogene mutation patterns (Figure 2d).

### 3.3 Microfluidic device for spheroids culture

**FIGURE 3:**
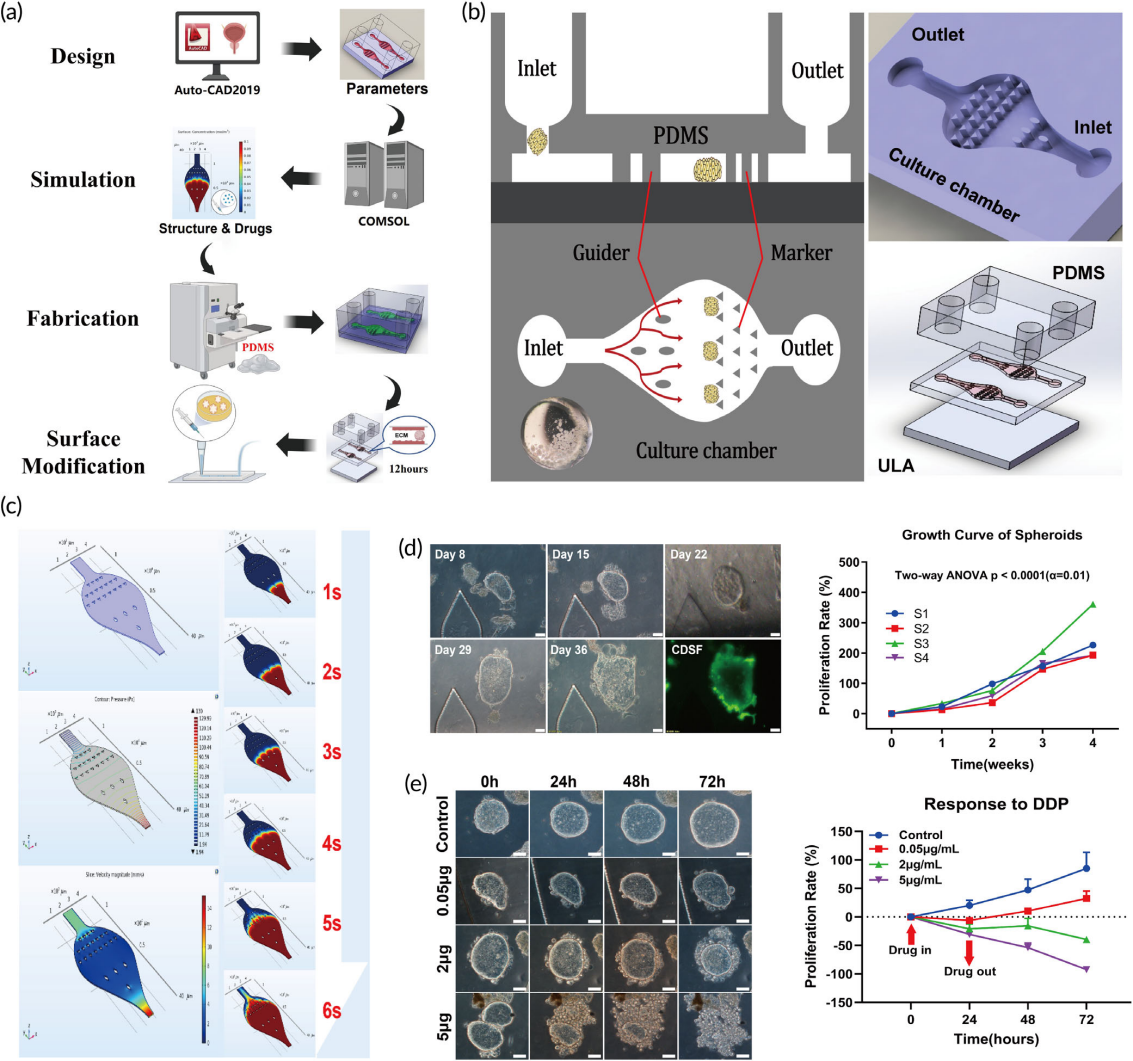
Microfluidic devices for culture tumor spheroids and drug sensitivity assay. (a) Schematic of the workflow for designing and constructing microfluidic devices for culture tumor spheroids. (b) Schematic of the design of microfluidic device. (c) Simulated drug concentration distribution in the microfluidic device. (d) Bright field and live cell staining of spheroids showing growth of tumor spheroids in microfluidic devices without passaging for more than 1 month (left) and the corresponding growth curve represented by percentage of area (right) *p* < 0.01 (two‐way ANOVA, scale bar, 50 μm). (e) Representative bright field images showing the morphological changes of tumor spheroids after treating with different concentrations of cisplatin (DDP) (scale bar, 50 μm).

Although spheroids could be cultured in vitro in ULA plates, we encountered certain disadvantages. Including loss of spheroids during medium exchange, difficulty in measuring the viability of each spheroid after drug treatment due to their changing position in the plates, and large number of spheroids required for drug sensitivity assay. Recently, the microfluidics has been investigated for spheroids formation and cultivation.^14^, ^20^ We, thus, developed a novel, simple, and low‐cost microfluidic device that could be utilized to culture spheroids by mimicking the actual condition of solid tumors in vivo, and to monitor the response of spheroids to chemotherapeutic agents. The design and parameters of this bladder‐shaped microfluidic device are illustrated in Figure 3a. In this design, the guiders section of the microfluidic device was composed of a series of streamlined guide columns that slow down the flow rate, reduce bubbles, and separate the introduced spheroids into different locations within the chamber. The trapping section consisted of triangle‐shaped columns to ensure proper entrapment of the spheroids while preventing them from flowing out of the chamber under hydrodynamic pressures induced by the flow, and to label the spheroids with marker on the triangle columns (Figures 3b and S2). We conducted simulation experiments to analyze the flow mechanics and drug concentration diffusion in the microfluidic device, which proved flow velocity and pressure were evenly distributed in the microfluidic chamber, and the drug diffused evenly and rapidly in the chip within 6 s (Figure 3c).

We found spheroids could be efficiently cultured in microfluidic device, with 0.5 mm^3^ piece of tumor tissue yielding enough spheroids to seed >12 microfluidic device (5–15 spheroids per device). Furthermore, we demonstrated spheroids could be successfully cultured in microchamber without passaging for over 4 weeks, which provided a suitable timeframe for real‐time monitoring of drug sensitivity, and different tumors showed varied growth rates (Figures 3d and S2E). Live cell staining revealed that proliferating cells were mainly confined to the periphery of the spheroids, simulating the condition of solid tumors in vivo, where cells at the core of the tumor may be quiescence or senescence due to lack of oxygen and nutrients.^21^ The main goal of culturing spheroids in the microfluidic device was to predict drug response as a preclinical model. As displayed in Figures 3e and S2F, spheroids showed varied responses (indicated by shrinkage of spheroids size) to different chemotherapeutic agents and concentrations. This microfluidic device can be used to simulate intravenous chemotherapy and intravesical instillation.

### 3.4 Drug sensitivity assay of bladder cancer spheroids

**FIGURE 4:**
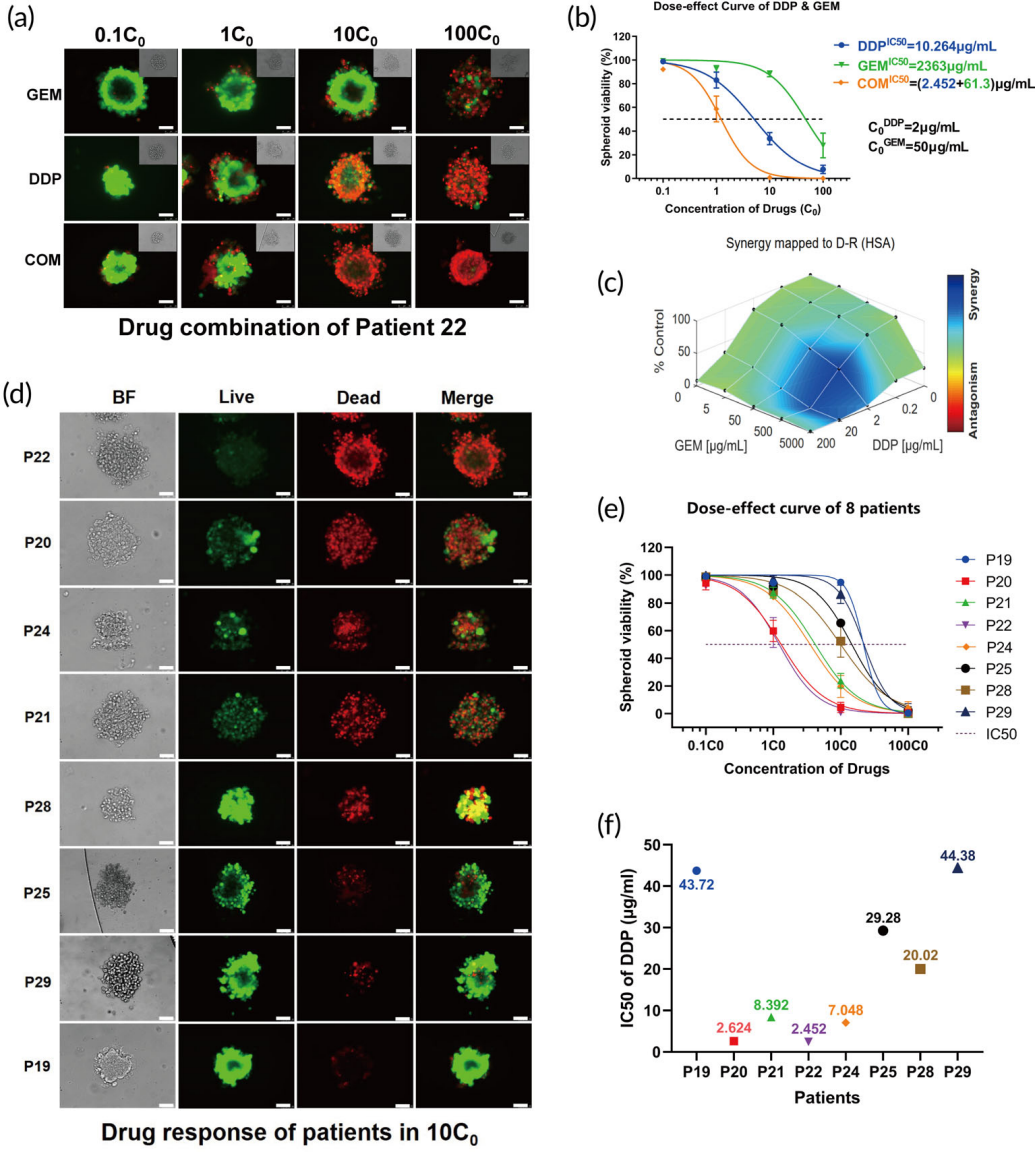
Tumor spheroids drug sensitivity assay in the microfluidic device. (a) Representative fluorescence images of spheroids stained with calcein‐AM (green, live cells; red, dead cells). Images were taken 24‐h after drug treatment with different drugs and concentrations. C_0_ represent the highest serum concentration of each drug (scale bar, 50 μm). (b) Dose response curves showed patients 22 spheroids treated with cisplatin (DDP), gemcitabine (GEM), and combination (DDP + GEM). (c) Drug–drug interaction between DDP and GEM analyzed using the Combenefit software. (d) Representative fluorescence images 24‐h after spheroids treated with DDP + GEM at concentration of 10C_0_ (scale bar, 50 μm), BF, bright field. (e) Dose response curves for tumor spheroids obtained from eight patients treated with DDP + GEM. (f) The IC50 of the DDP for the eight patients.

According to clinical practice, we tested the drug sensitivity of spheroids to standard neoadjuvant/adjuvant chemotherapeutic regimen (GC). We used a live/dead cell double staining kit to measure cell viability within the microfluidic device. The maximum plasma concentrations of cisplatin and gemcitabine obtained from pharmacokinetics studies were used as reference (C_0_, 2 μg/mL for cisplatin and 50 μg/mL for gemcitabine),^22^, ^23^ and spheroids were treated with different multiples of the C_0_ values (0.1, 1, 10, and 100 × C_0_, as shown in Figure 4a). As approximately 80% cisplatin and 100% gemcitabine were excreted or eliminated within 24 h after administration, spheroids were treated with drugs for 24 h.^22^, ^23^ The dose‐dependent response curve of spheroids after treatment was shown in Figure 4b, where the spheroids from P22 were resistant to gemcitabine with IC50 of 2363 μg/mL, and relatively sensitive to cisplatin with IC50 of 10.26 μg/mL. The combination of GC enhanced the antitumor activity, as indicated by a significant left shift of the dose–response curve. The interaction between cisplatin and gemcitabine was evaluated by a combination index, which suggested a synergistic effect (Figure 4c). We further used this preclinical model to test the sensitivity of cisplatin and gemcitabine regimen on tumors from 12 patients(P19‐P30), of which eight patients had successful drug sensitivity assay (Figures 4d and S3C). The other four failed due to tissue crumble (P23, P26, P27, and P30) and flowed through microfluidic devices. The sensitivity of the chemotherapy regimens varied significantly between spheroids of different patients. The spheroids of P20 and P22 were more sensitive to GC regimen, whereas P19 and P29 were more resistant, other spheroid lines showed partial response to the combination chemotherapy (Figure 4e,f). We found the viability of spheroids after treatment with 10 times the reference concentration (10C_0_) was highly correlated with the IC50 (Figure S3D,E). It suggested that the drug sensitivity assay method could be simplified by measuring the viability of spheroids under 10C_0_, especially when insufficient spheroids could be obtained from tumor samples for drug sensitivity tests at different concentrations.

### 3.5 Spheroids' drug responses comparison with PDX models

**FIGURE 5:**
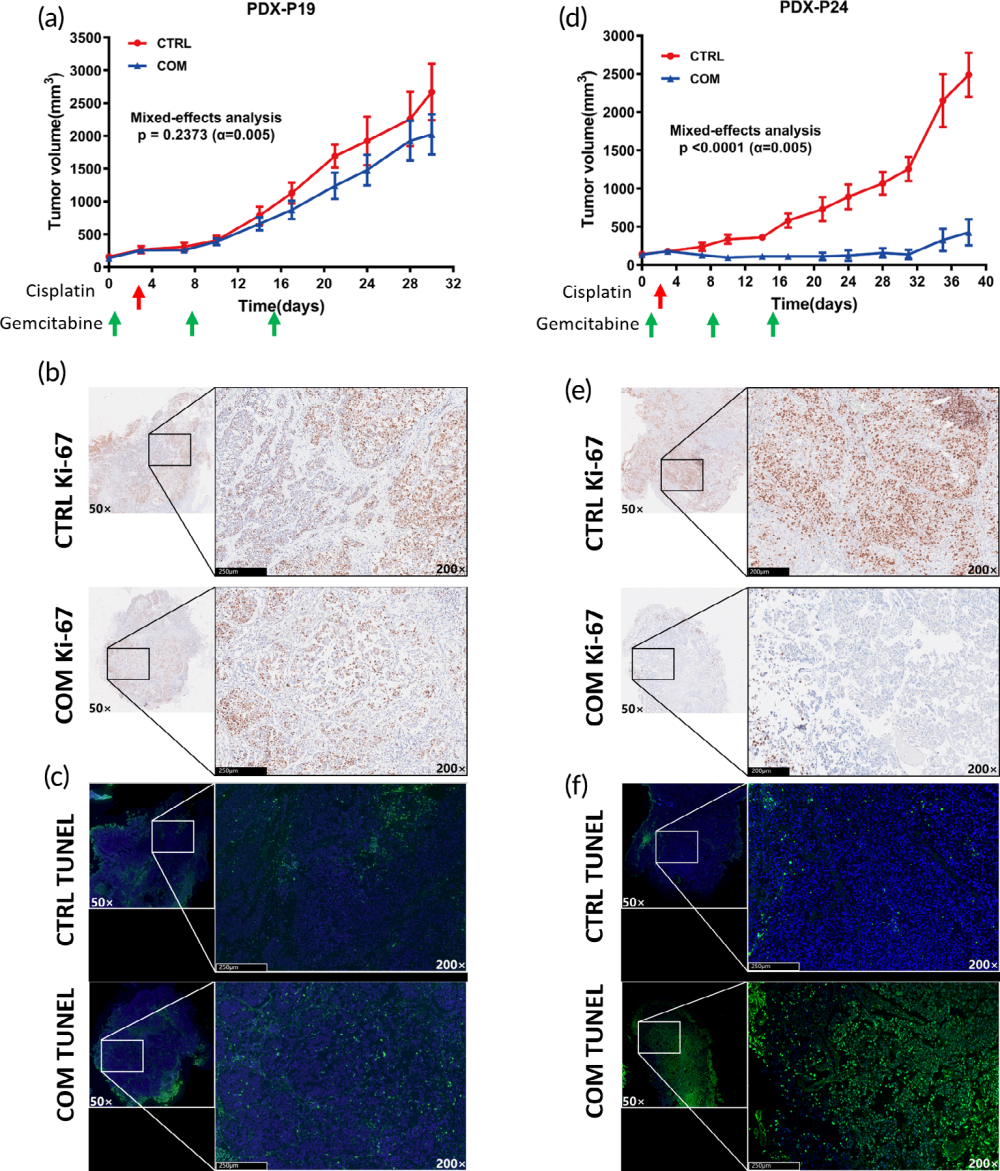
Validation of drug response in patient‐derived xenografts (PDX). (a) Tumor growth curve of PDX derived from patient 19 (PDX‐19). Tumors treated with cisplatin (DDP) and gemcitabine (GEM) grew slightly slower than the control group (CTRL), *p* = 0.24. (b) Immunostaining for Ki‐67 in PDX tumor 3 days after the last dose of GEM treatment, Ki‐67 expression was slightly reduced in the treatment group (scale bar, 250 μm). (c) TUNEL assay for PDX tumor 3 days after the last dose of GEM treatment, TUNEL‐positive cells in the treatment group were slightly more than CTRL group (scale bar, 250 μm). (d) Tumor growth curve of PDX derived from patient 24 (PDX‐P24). Tumors' growth rate was significantly inhibited by the combination treatment. (e) Immunostaining for Ki‐67 in PDX tumor 3 days after the last dose of GEM treatment, Ki‐67 expression was significantly reduced in the treatment group (scale bar, 250 μm). (f) TUNEL‐positive cells in the treatment group were significantly more than CTRL group (scale bar, 250 μm).

To determine whether spheroids successfully mimicked the response to therapy in vivo, we compared the in vitro drug responses with matched PDX models. Tumor tissues from four patients, whose spheroids responded differently to the GC regimen, were transplanted subcutaneously into NSG mice to establish PDX models (Figure S4A,B). Two PDX models from patient 19 and patient 24 were successfully established. When tumors reached ~150 mm^3^, they were treated with one cycle of GC regimen or saline control. The dose‐response curve of spheroids suggested that P19 was more resistant to GC regimen than P24 (Figure S4C). The matched PDX‐P19 model showed that tumor growth was slightly retarded by the GC regimen (Figure 5a) with the median time to achieve a tumor volume of 10 times the baseline at 20 days and 21 days in control and GC treatment groups, respectively (*p* = 0.49, Figure S4D). Ki‐67 and TUNEL staining suggested that the cellular proliferation was slightly inhibited and apoptotic cells increased by chemotherapy (Figure 5b,c). In contrast, the GC regimen significantly delayed the tumor growth in the PDX‐P24 model (Figure 5d). We also found a significant decrease in Ki‐67‐positive cells and an increase in TUNEL‐positive cells after GC regimen (Figure 5e,f). These results indicated that the drug responses observed on microfluidic device‐based spheroids could be recapitulated in vitro and in vivo.

### 3.6 Clinical correlation with spheroid‐based drug sensitivity assay

**FIGURE 6:**
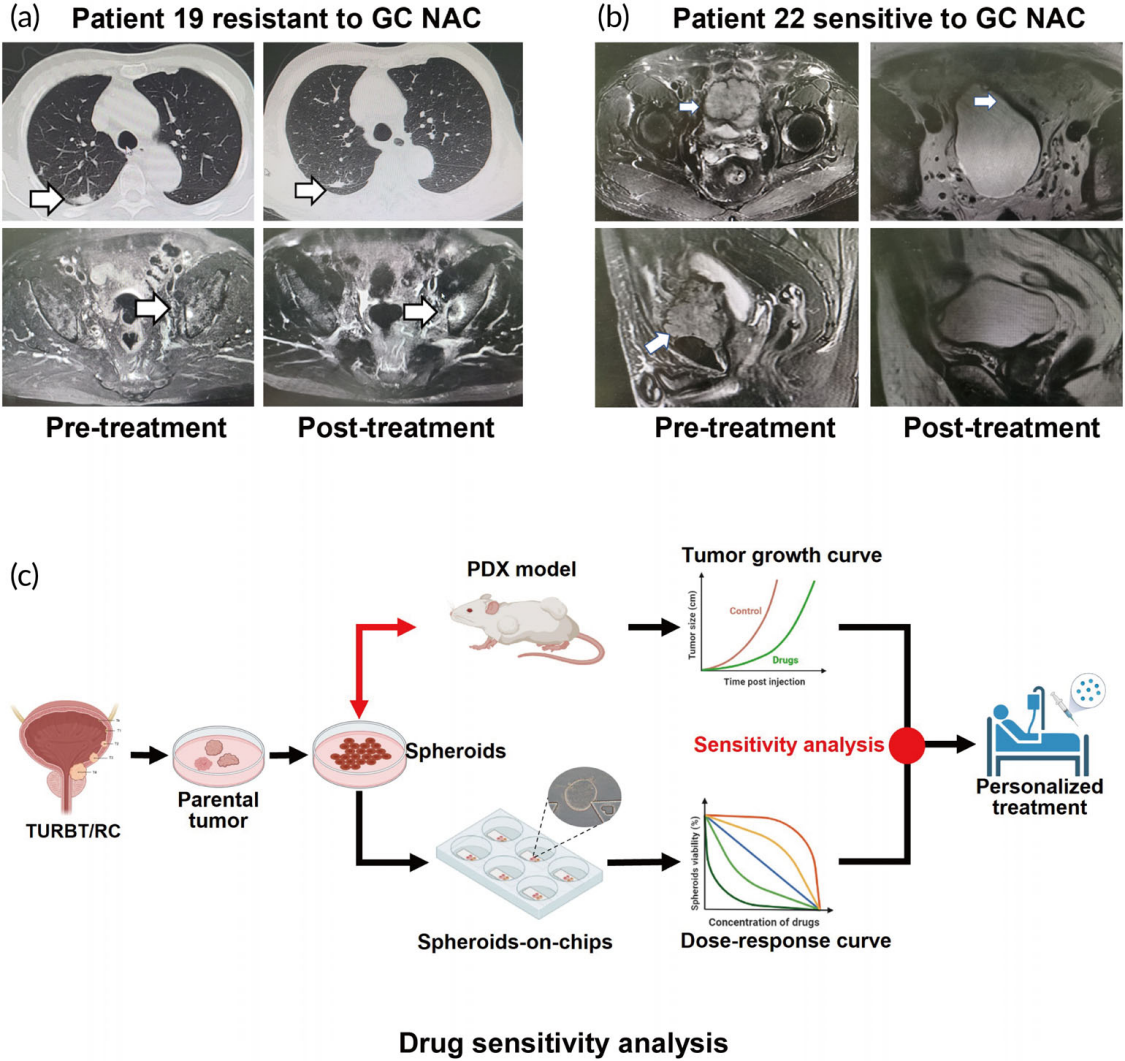
Spheroids drug sensitivity assay correlated with clinical response. (a) Patient 19 had primary bladder cancer and suspicious lesions were detected at lung and left iliac bone at initial diagnosis. After two cycles of cisplatin and gemcitabine neoadjuvant chemotherapy, lung lesion was stable, but the left iliac lesion progressed. (b) Patient 22 had extravesical tumor recurrence after transurethral resection of bladder tumor, and this patient had complete response after cisplatin and gemcitabine neoadjuvant chemotherapy. (c) Simplified scheme of using tumor spheroids drug sensitivity assay to guide personalized treatment for bladder cancer patients.

We found spheroids from different patients exhibited varying sensitivities to the tested drugs. To understand the relationship between spheroids drug sensitivity and clinical response, follow‐up information was obtained from patients with spheroids drug sensitivity assay results. The baseline characteristics of patients are shown in Figure S5A. Three patients received neoadjuvant GC chemotherapy and two patients received adjuvant GC chemotherapy. The detailed treatment information is shown in Figure S5B. Two patients with metastasis or recurrence were evaluated for their response to GC regimen through radiological imaging. Computerized tomography evaluation of patient 19's tumor displayed resistance to the GC treatment as there was no significant change in the lung lesion and an enlargement of the bone metastasis lesion (Figure 6a). On the other hand, patient 22 exhibited a complete response to GC regimen (Figure 6b). The clinical drug response of patients who received the GC regimen was predicted based on the in vitro drug‐sensitivity response of their corresponding spheroid models for GC regimen (Figure 4e). These findings indicated that the spheroids model has the potential to predict clinical drug sensitivity of bladder cancer patients (Figure 6c). However, the correlation between spheroids drug sensitivity assay and clinical response must be verified in a bigger cohort of patients.

## 4 DISCUSSION

Given the obvious heterogenicity of cancers, therapeutic strategies against cancer have experienced a shift from broad cytotoxic drugs to more personalized targeted treatment over the past decades.^24^ In terms of bladder cancer, cisplatin‐based combination chemotherapy remains the standard treatment for advanced‐stage bladder cancer since the 1980s. However, the overall survival is around 9–15 months, and approximately 50% of cisplatin‐eligible patients do not respond to the treatment. Recently, immune checkpoint inhibitors emerged as an alternative first‐line option for patients who are not suitable for platinum‐based chemotherapy and as second‐line treatment for patients who failed platinum‐based chemotherapy.^1^ To improve the effectiveness of chemotherapy and facilitate clinical decision‐making, cisplatin‐based chemotherapy agents sensitivity assay before clinical treatment would be of significant value. It contributes to the selection of the appropriate regimen for each patient and individualized treatment.

In the present study, we established the bladder tumor spheroids model to predict the sensitivity of chemotherapeutic agents. The spheroids model recapitulated the histopathological and molecular features of their corresponding parental tumors and could be an ideal candidate for drug sensitivity assay. Unlike organoids, spheroids models mimic the solid tumor microenvironment, including tumor cells and stromal components such as fibroblasts, and epithelial and immune cells. We developed a simple and efficient approach to establish bladder cancer patient‐derived spheroids. Interestingly, we found spheroids of a certain number and size could maintain growth in regular ECM medium without the addition of several cytokines and growth factors that were typically required for sustaining organoid growth, such as fibroblast growth factor, epidermal growth factor, and Nicotinamide.^10^, ^25^, ^26^ This could be explained that the interactions still exist between stromal cells and cancer cells through the secretion of cytokines and growth factors, which promote events such as tumor proliferation, invasion, as well as therapeutic resistance.^14^ However, specific autocrine signals from stromal cells contributing to the bladder cancer cells in the spheroids remained to be elucidated in the future study.

Microfluidic technology enables the manipulation of fluids at the submillimeter scale, allowing for the miniaturizing of traditional laboratory operations in biology and chemistry, such as sample preparation, reaction, and assays.^12^ Microfluidic technology overcomes the limitations of traditional in vitro drug sensitivity assay, which lacks sufficient resolution or is unable to sustain a steady and reliable drug concentration gradient over time.^27^ We hypothesized that the application of microfluidic technologies to spheroids culture could offer advantages, such as reduced sample requirements, lower drug consumption, ease of manipulation, and real‐time monitoring of drug sensitivity. We, thus, successfully designed a microfluidic device for spheroids culture and drug sensitivity assays. The spheroids could grow in the microfluidic device without developing necrosis for more than 30 days, providing a sufficient timeframe for studying drug sensitivity. By utilizing this microfluidic system, we could also culture spheroids with high efficiency. As a result, drug sensitivity assay for the commonly used cisplatin‐based regimen could be performed rapidly and conveniently. Spheroids derived from different patients usually had varying components of stromal cells as well as different sizes or shapes, which can affect the therapeutic response and IC50 value.^28^ It was revealed that the penetration ability of drugs in spheroids was dependent on spheroids' size, known as a limited mass transport effect.^29^ Therefore, determining the drug sensitivity of spheroids from different patients by directly comparing the IC50 value might not be accurate. We also found that the viability of spheroids after the GC regimen with 10 times the maximum plasma concentration highly correlated with the IC50, which could help us to determine drug sensitivity. For example, at 10C_0_ concentration, patient 19 exhibited 9% spheroid viability inhibition, whereas patient 22 showed 96% inhibition compared with the corresponding controls (Figure S3D). This suggests that patient 22 was more sensitive to GC treatment compared with patient 19, which aligns with the clinical results. Although the clinical response of two patients who received GC adjuvant chemotherapy mirrored the drug‐sensitivity response of their corresponding spheroids models, the threshold of drug sensitivity assay to predict clinical response is currently unknown and requires determination with a larger sample size of patients who have undergone chemotherapy.

Preclinical cancer models that resemble the complexity and heterogeneity of tumors are essential for drug development and sensitivity assay. Currently, two‐dimensional cell culture and PDX models remain predominant preclinical models, but the derivation of cell lines, as well as PDX, is inefficient, time‐consuming, and labor‐intensive, limiting their contribution to personalized treatment on a broad scale.^10^, ^11^ Recent advancements in three‐dimensional tumor cell culture systems, termed organoids, have shown promise as preclinical model for studying biological functions and drug discovery because it partially recapitulates the complexity and function of solid tumors in vitro.^11^ However, organoids fail to fully reconstruct the tissue microenvironments, which play a critical role in tumor development, progression, and drug response.^30^, ^31^ So far, organoids have been successfully generated for a wide range of cancer types, including lung,^32^ colorectal,^33^ gastrointestinal,^34^ liver,^6^ breast,^10^ prostate,^35^ pancreatic,^36^ and urothelial,^26^ allowing tumor evolution and drug response studies. In terms of UC, organoids for bladder cancer and upper UC have been developed.^9^, ^26^ It was revealed that UC organoid lines changed their basal or luminal subtypes during culture due to the absence of tumor microenviroment (TME). These organoids often reverted to their original phenotype when transplanted into mice.^9^, ^26^ Kim et al.^31^ developed bladder tumor assembloids by reconstitution of bladder tumor organoids with cancer‐associated fibroblasts and demonstrated that luminal to basal drift in tumor subtype could thus be prevented, suggesting that proper stromal cells and signals might be needed to maintain the tumor phenotype. However, coculture systems like these are complex, inconsistent, and lack a standardized protocol or guidelines specifying the cell types to incorporate.^13^

It is well known that solid tumors have a heterogenic cellular composition, including not only cancer cells but also TME which consists of stromal cells, immune cells, extracellular matrix, and secreted factors. Analyzing tumor tissue containing TME is necessary for valuable drug screening.^30^, ^37^ Lee et al.^38^ demonstrated that the cocultivation of the cancer cells with cancer‐associated fibroblasts improved resistance to drugs. Kim et al.^31^ also found that therapeutic responses of bladder tumor assembloids to chemotherapeutic drugs were decreased compared with conventional bladder tumor organoids. Unfortunately, most drug sensitivity tests overlook the influence of stromal cells on the tumor cells; therefore, the cellular physiology and response to the tested drugs may be greatly impeded.^12^, ^31^ As a result, we developed a microfluidic platform on which bladder cancer spheroids, containing both tumor and stromal cells, were cultured for drug sensitivity assay, and this might provide guidance for individualized treatment.

Limitations of the present study need to be acknowledged. First, the lack of immune system of our culture platform might potentially affect the outcome of drug sensitivity assay and restrict its utility in testing the sensitivity of immune checkpoint inhibitors. Second, the passage of spheroids relies on PDX, which is time‐consuming. In addition, the persistent presence of murine viruses in PDX models and the replacement of TME with murine stromal components might alter the tumor sensitivity to treatment regimens.^39^ Third, although spheroids obtained from a small volume of fresh tissue was enough for testing common chemotherapy agents for bladder cancer, it was not possible for high‐throughput drug screening. Fourth, the current microfluidic device is low throughput, and there is a lack of standardized assays for spheroids imaging, quantification, and automated analysis for drug screening. Efforts are being made to improve the drug sensitivity assay platform by fabricating a microfluidic device with gradient generators with the microfluidic device and make sensitivity analysis become high throughput and efficient by introducing artificial intelligence. Fifth, the threshold of spheroids sensitivity assay to predict the actual response from patients still needs to be observed in a larger sample size.

In summary, we successfully established bladder cancer spheroids recapitulating histological and genetic features of solid tumors. We further designed and fabricated a microfluidic device to efficiently test the drug sensitivity of spheroids to the standard GC regimen for bladder cancer, and the outcomes demonstrated a strong correlation between PDX models and clinical response in patients. Our results indicated that drug sensitivity assay of bladder cancer spheroids has the potential to guide the selection of personalized chemotherapy regimens for patients.

## AUTHOR CONTRIBUTIONS

**Yidie Ying:** Data curation (equal); investigation (equal); visualization (equal); writing – original draft (equal). **Qiao Xiong:** Conceptualization (equal); formal analysis (equal); investigation (equal); methodology (lead); visualization (equal); writing – original draft (equal). **Ting Liu:** Conceptualization (equal); data curation (equal); methodology (equal); resources (equal); software (equal); validation (equal); writing – original draft (equal). **Xiaowen Yu:** Data curation (equal); formal analysis (equal); investigation (equal); writing – original draft (equal). **Ziwei Wang:** Data curation (equal); software (equal); validation (equal); visualization (equal); writing – original draft (equal). **Hongliang Gao:** Formal analysis (equal); investigation (equal); resources (equal); writing – original draft (equal). **Tianhai Lin:** Data curation (equal); formal analysis (equal); investigation (equal); visualization (equal); writing – original draft (equal). **Weihua Fan:** Methodology (equal); resources (equal); software (equal); visualization (equal); writing – original draft (equal). **Zhensheng Zhang:** Conceptualization (equal); project administration (equal); supervision (equal); writing – original draft (equal); writing – review and editing (equal). **Qiang Wei:** Project administration (equal); resources (equal); supervision (equal); writing – original draft (equal); writing – review and editing (equal). **Yuqing Ge:** Conceptualization (equal); funding acquisition (equal); project administration (equal); software (equal); supervision (equal); writing – original draft (equal); writing – review and editing (equal). **Shuxiong Zeng:** Conceptualization (equal); funding acquisition (equal); methodology (equal); project administration (equal); writing – original draft (equal); writing – review and editing (equal). **Chuanliang Xu:** Conceptualization (equal); funding acquisition (equal); project administration (equal); supervision (equal); writing – review and editing (equal).

## FUNDING INFORMATION

This research was financed by grants from National Natural Science Foundation of China ( 82172871, 81972391, and 82272950), Shanghai Municipal Health Commission (2022YQ010), National Key R&D Program of China (No. 2022YFA1104703), Qihang Program of Naval Medical University (2021), Discipline Development Plan of Changhai Hospital (2019YXK041), Science and Technology Commission of Shanghai Municipality (20Y11904800, 22140903700, and 23XTCX00700).

## CONFLICT OF INTEREST STATEMENT

The authors declare no conflicts of interest.

### PEER REVIEW

The peer review history for this article is available at https://www.webofscience.com/api/gateway/wos/peer-review/10.1002/btm2.10624.

## Supporting information


**Data S1:** Supporting Information.

**Figure S1:** Morphological features of tumor spheroids cultured in the petri dishes. (A) Standard flowchart for preparation of tumor spheroids. (B) Morphological features of spheroids cultured in ultralow attachment (ULA) and tissue culture treated (TCT) dishes, spheroids could maintain the spheroid feature in ULA dishes while tend to adhere and expand in the TCT dishes. (C) Low‐grade spheroids tended to be more adhesive and appeared to be flat on the ultralow‐attachment plates than high‐grade. (D) H&E staining of spheroids at >100 μm and 70–100 μm diameter, larger spheroids prone to form necrotic core. (E) Representative morphological images of the spheroids from each patient. (F) H&E staining of parental tumor tissue, tumor spheroids and spheroids cultured from the corresponding xenograft tumor in NSG mice.

**Figure S2:** Design microfluidic devices for culture tumor spheroids and drug sensitivity assay. (A) The architecture of the microfluidic device. (B) Parameters of each microfluidic device component. (C) The workflow of constructing a microfluidic device. (D) Effect of different surface modification methods of microfluidic device on spheroids culture. (E) Tumor spheroids obtained from different patients showing distinct growth rate in the microfluidic device. (F) Stimulation of drug sensitivity assay for intravesical instillation (pirarubicin, THP), single chemotherapeutic agent (cisplatin, DDP; gemcitabine, GEM) and combinational treatment (DDP + GEM).

**Figure S3:** Establishment of drug sensitivity assay based on tumor spheroids in the microfluidic devices. (A) Fluorescence images of negative and positive control. (B) Standard process and viability calculation formula of fluorescence images, S^NC^, negative control, S^PC^, positive control, S^L^ live cells after treatment, S^D^, dead cells after treatment. (C) Representative fluorescence images of spheroids (green, live cells; red, dead cells) treated with different concentrations of cisplatin (DDP) and gemcitabine. Images were taken 24‐hour after drug treatment. C0 represent the highest serum concentration of each drug. (D) Spheroids viability measured at the concentration of 10 C0, and this was highly correlated with the IC50 of cisplatin (E). BF, bright filed.

**Figure S4:** Validation of drug response in patient derived xenografts (PDX). (A) Correspondence between spheroids, PDX model. (B) Schematic drawing comparing the drug sensitivity assay timeline between PDX model and tumor spheroids in the microfluidic device. (C) Dose response curve of tumor spheroids obtained from patient 19 and 24, spheroids were treated with cisplatin and gemcitabine. (D) The median time of the tumor growth to 10 times the baseline in the control and combination treatment group in PDX‐P19and PDX‐P24. (E, F) Body weight of mice was slightly decreased in combination treatment groups.

**Figure S5:** Detailed information of patients used for spheroids' drug sensitivity assay. (A) Baseline characteristics of patients for spheroids' drug sensitivity assay. Information was collected when the tumor samples were obtained from surgery. (B) Time chart of each patient's diagnosis and treatment (the starting point was the time of operation at which the sample was collected). TURBT, transurethral resection of bladder tumor; RC, radical cystectomy; RNU, radical nephroureterectomy; GC, gemcitabine and cisplatin or carboplatin; ICIs, immune checkpoint inhibitors.

## Data Availability

The data for this study are available within the article, with additional data available in the Supporting Information.
