# Supplementary material for: Establishment of bladder cancer spheroids and cultured in microfluidic platform for predicting drug response
Source: Bioeng Transl Med. 2023 Dec 4;9(2):e10624. doi: 10.1002/btm2.10624 (PMC10905551; doi:10.1002/btm2.10624)
Supplement: Supplementary file 1 — Data S1: Supporting Information. [file BTM2-9-e10624-s002.docx]

**Microfluidic device Manufacture**

The microfluidic device designed and manufactured on the basis of polydimethylsiloxane (PDMS) has the characteristics of hydrophobic and breathable, which enable a wide range of biochemical and clinical applications^1,2^. In this study, we design and fabricated a kind of microfluidic device to culture tumor spheroids in vitro. The flowchart of microfluidic manufacture including 4 main steps was shown in **Figure 3A**. Here we present the four steps of device design, simulation, fabrication and surface modification in more detail and all of the materials were shown in figures and supplementary figures.

**Design**

Based on structural resistance characteristics of underwater streamlined body and bladder shape, the microfluidic device was design as a hydrodynamic structure to mimic human bladder by using AutoCAD software (Autodesk AutoCAD 2019, Autodesk, San Rafael, CA, USA). Design schematic and parameter table were shown in **Figure S2A** and **S2B**. Exactly, the microfluidic devices were fabricated by bonding a breathable PDMS layer to the ultra-low attachment culture dishes. The PDMS layer contained a hydrodynamic microchamber with 50 μm height, 4 drop-shaped guiders, 19 triangular markers, and two different sizes of flow channels. The design was selected in a way that any introduced spheroids would be trapped and marked within the chamber (**Figure 3B**).

**Simulation**

To adjust the device parameters and structure for a more stable state, we used software COMSOL Multiphysics 5.6 (COMSOL AB, Stockholm, Sweden) and related formulas for simulation analysis^3,4^. Fluid pressure and drug concentration parameters were set according to experimental and clinical practice. We analyzed the flow mechanics and drug concentration diffusion through simulation experiments several times to get a better model. And the last analysis shown that flow velocity and pressure were evenly distributed in the microfluidic chamber, and drug diffused evenly and rapidly in the chip within 6 seconds, which proved the device was well worked (**Figure 3C**).

**Fabrication**

The microfluidic chip was fabricated applying standard soft lithography and replica molding of PDMS (Dow Corning, US) (**Figure S2C**). In brief, a photoresist (SU-8 3050 Microchem, USA) was applied to the silicon wafer and patterned by using a photomask and UV light, obtaining a positive channel geometry wafer. Subsequently, a PDMS mixture of silicone elastic matrix and a curing agent at a weight ratio of 10: 1 was poured onto a silicon wafer after degassing with a vacuum desiccator (Yili Botong, China)^5^. And then, the mixture was utterly cured in an oven at 70 °C for 4 hours. Repeat the pouring steps to form the upper PDMS layer. After carefully removing it from the template, this PDMS was cut into equal-sized bricks and backup. The contact surfaces of the PDMS layer were plasma treated by a plasma sputtering coater (Hengming Company, China) for 60 seconds, and then they were immersed in 5% (3-aminopropyl) triethoxysilane (APTES) and 1% 3-Glycidoxypropyltrimethoxysilane (GLYMO) aqueous solution for 20 minutes, respectively. Subsequently, the PDMS bricks were carefully aligned to the bottom of ultra-low attachment culture dishes after drying with nitrogen and heated at 65℃ overnight to ensure complete bonding.

**Surface Modification**

Before spheroids seeding, the microfluidic devices were sterilized by UV for 30 minutes, and the channels and chambers were incubated with different mediums to create a more suitable environment for spheroids culturing^6^. There mediums including ECM, alcohol and Polylysine were tested for about 12 hours in this step and the results shown that ECM medium was the better one (**Figure S2D**).


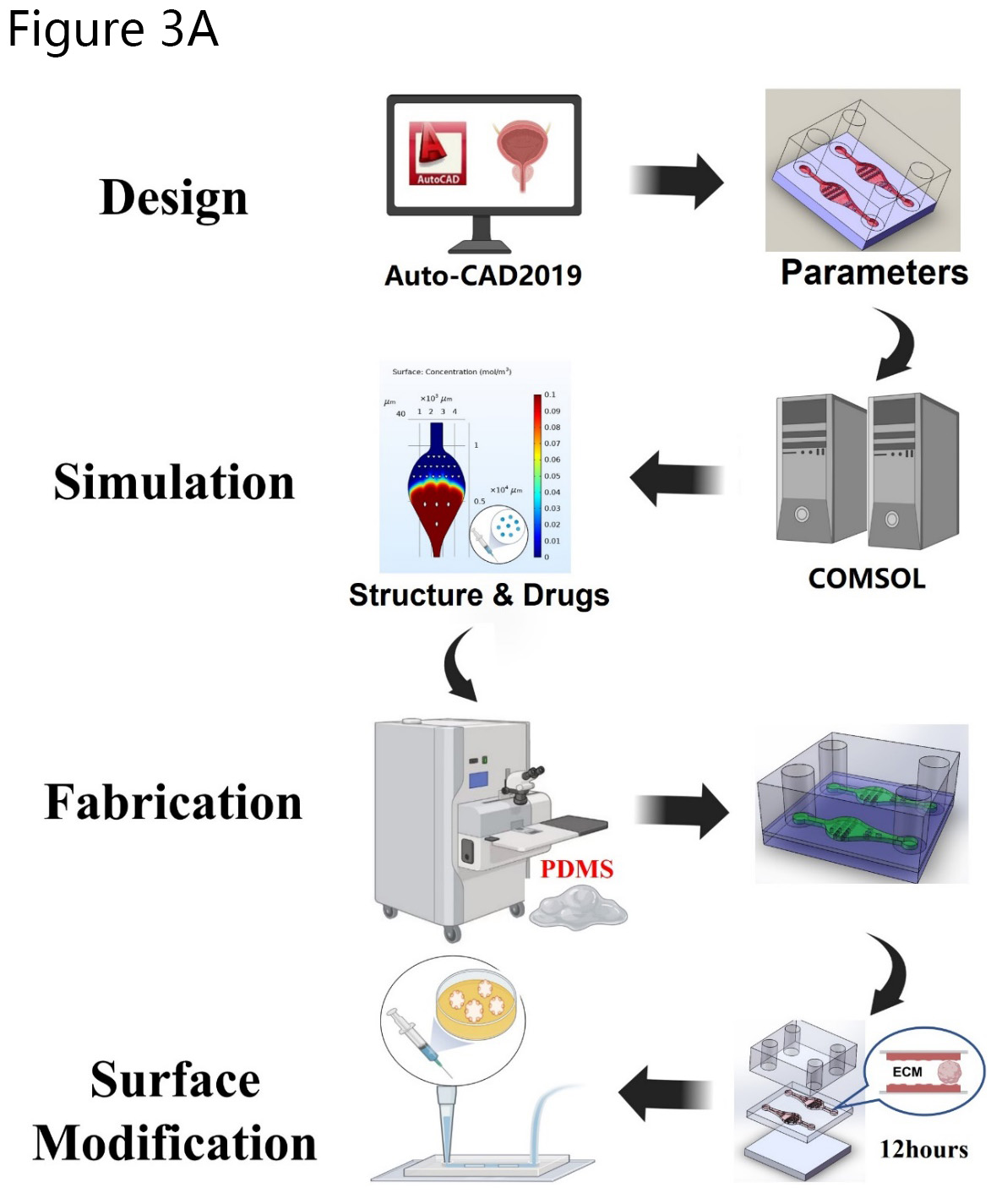


**Fig 3A. Flowchart of microfluidic device manufacture**


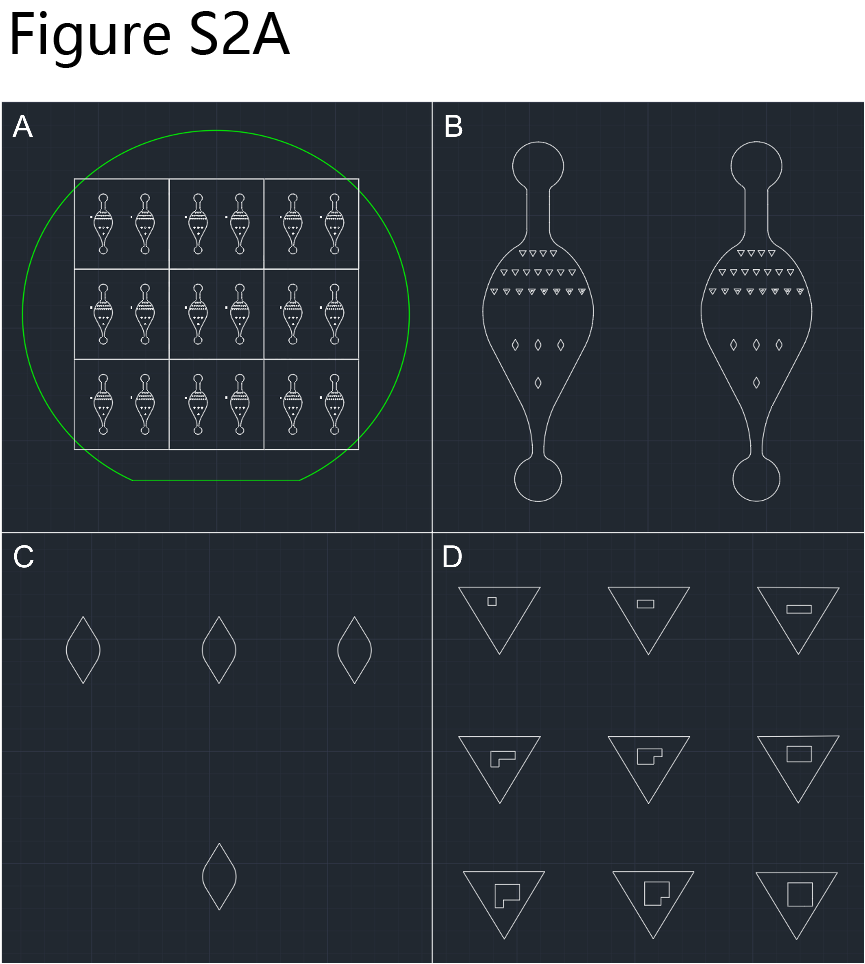


**Fig S2A. Design schematic of microfluidic device**


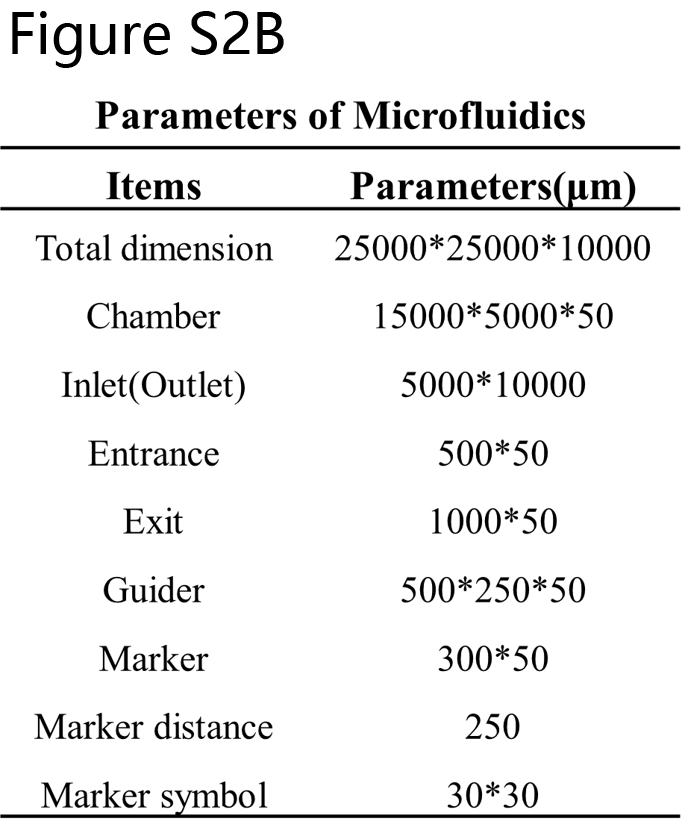


**Fig S2B. Design parameters of microfluidic device**


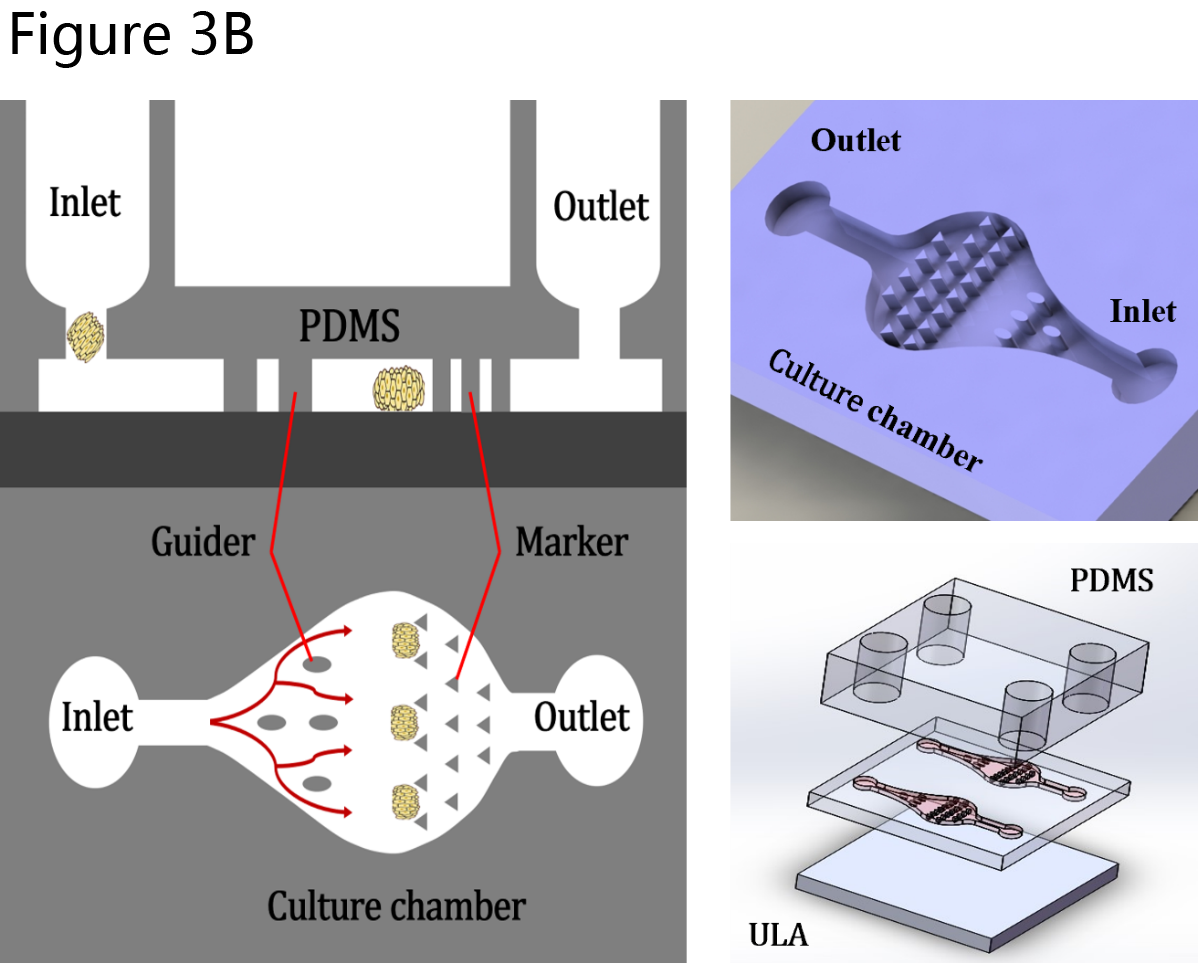


**Fig 3B. Design renderings of microfluidic device**


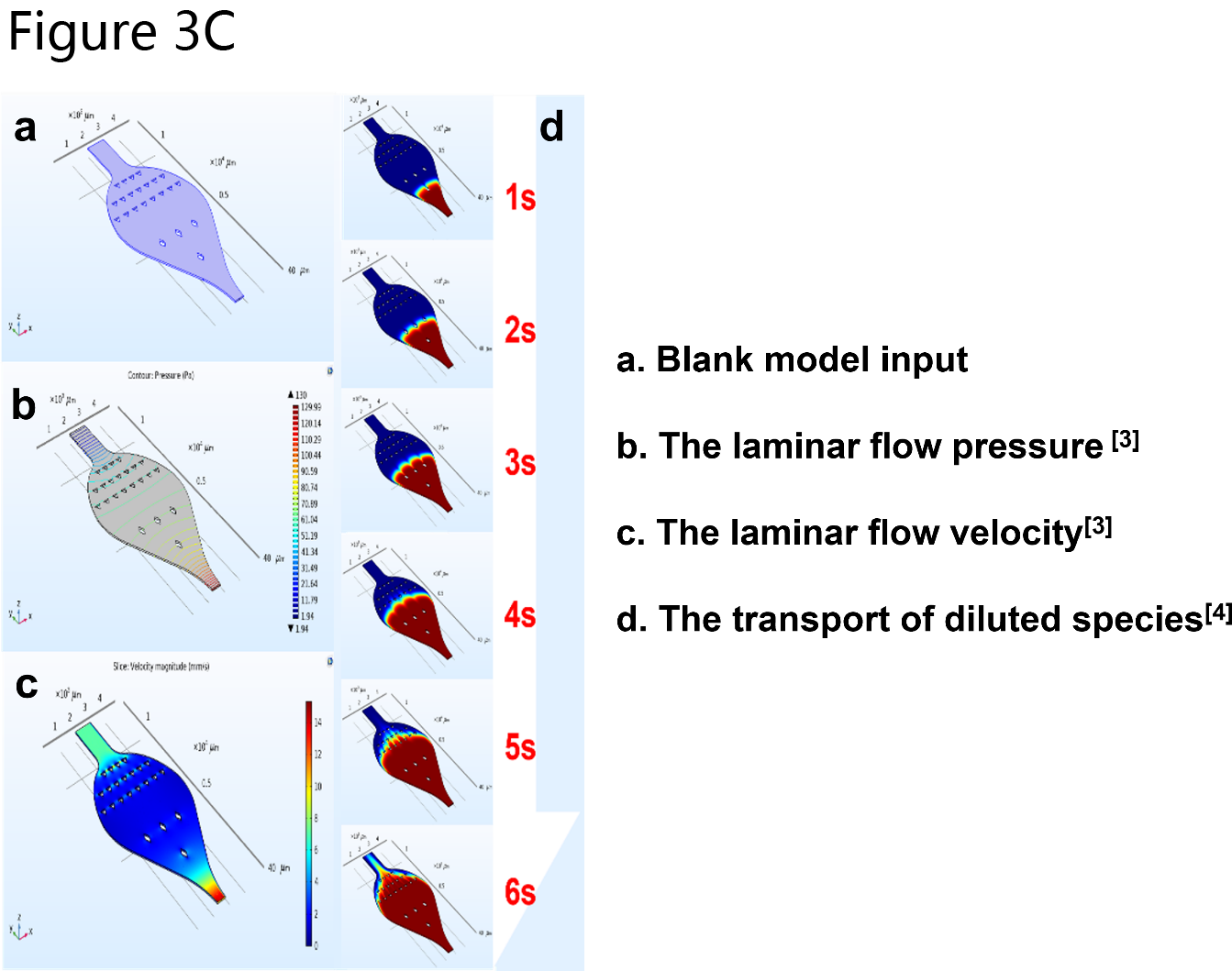


**Fig 3C. Simulation analysis of microfluidic device**


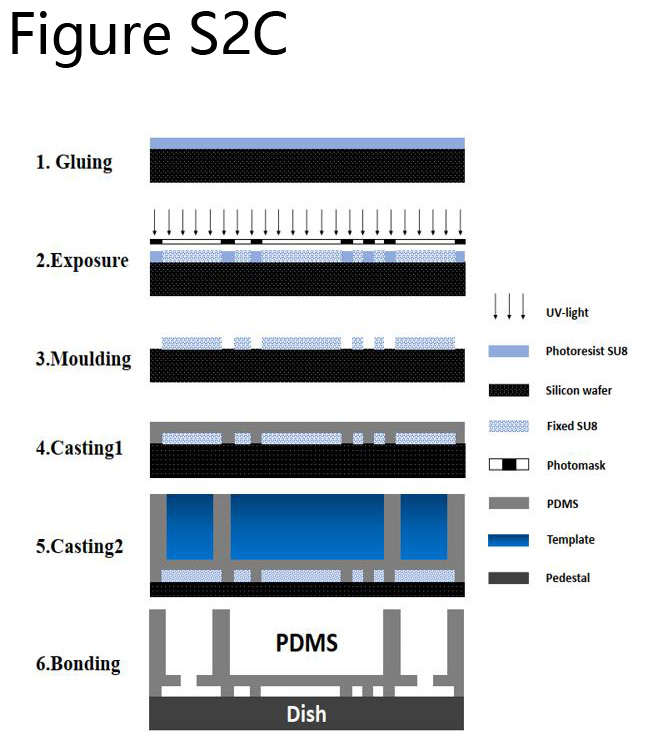


**Fig S2C. Fabrication process of microfluidic device**


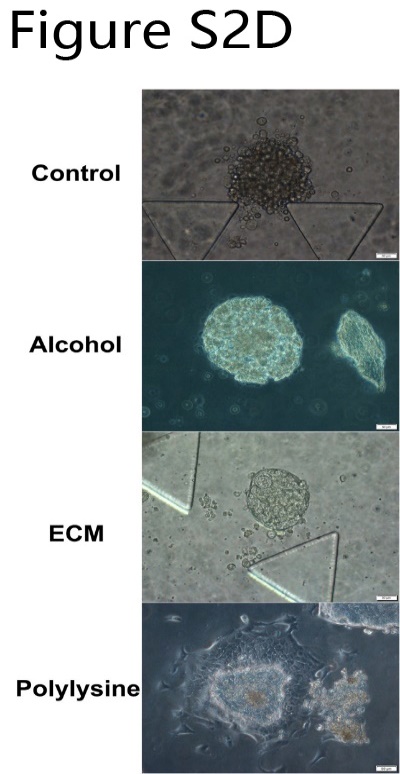


**Fig S2D. Surface Modification of microfluidic device**

**Reference:**

1. Amin R, Knowlton S, Hart A, et al. 3D-printed microfluidic devices. *Biofabrication*. Jun 20 2016;8(2):022001. doi:10.1088/1758-5090/8/2/022001

2. Jenkins RW, Aref AR, Lizotte PH, et al. Ex Vivo Profiling of PD-1 Blockade Using Organotypic Tumor Spheroids. *Cancer discovery*. Feb 2018;8(2):196-215. doi:10.1158/2159-8290.Cd-17-0833

3. Fraaije J, van Male J, Becherer P, Serral Gracià R. Calculation of Diffusion Coefficients through Coarse-Grained Simulations Using the Automated-Fragmentation-Parametrization Method and the Recovery of Wilke-Chang Statistical Correlation. *Journal of chemical theory and computation*. Feb 13 2018;14(2):479-485. doi:10.1021/acs.jctc.7b01093

4. Saqr KM. Wall shear stress in the Navier-Stokes equation: A commentary. *Computers in biology and medicine*. Mar 2019;106:82-83. doi:10.1016/j.compbiomed.2019.01.012

5. Dabaghi M, Saraei N, Fusch G, et al. An ultra-thin, all PDMS-based microfluidic lung assist device with high oxygenation capacity. *Biomicrofluidics*. May 2019;13(3):034116. doi:10.1063/1.5091492

6. Wang X, Agasid MT, Baker CA, Aspinwall CA. Surface Modification of Glass/PDMS Microfluidic Valve Assemblies Enhances Valve Electrical Resistance. *ACS applied materials & interfaces*. Sep 18 2019;11(37):34463-34470. doi:10.1021/acsami.9b12342

**Supplementary figure legends:**

**Figure S1. Morphological features of tumor spheroids cultured in the petri dishes. A** Standard flowchart for preparation of tumor spheroids. **B** Morphological features of spheroids cultured in ultra-low attachment (ULA) and tissue culture treated (TCT) dishes, spheroids could maintain the spheroid feature in ULA dishes while tend to adhere and expand in the TCT dishes. **C** Low-grade spheroids tended to be more adhesive and appeared to be flat on the ultralow-attachment plates than high-grade. **D** H&E staining of spheroids at >100 μm and 70-100 μm diameter, larger spheroids prone to form necrotic core. **E** Representative morphological images of the spheroids from each patient. **F** H&E staining of parental tumor tissue, tumor spheroids and spheroids cultured from the corresponding xenograft tumor in NSG mice.

**Figure S2. Design microfluidic devices for culture tumor spheroids and drug sensitivity assay. A** The architecture of the microfluidic device. **B** Parameters of each microfluidic device component. **C** The workflow of constructing a microfluidic device. **D** Effect of different surface modification methods of microfluidic device on spheroids culture. **E** Tumor spheroids obtained from different patients showing distinct growth rate in the microfluidic device. **F** Stimulation of drug sensitivity assay for intravesical instillation (pirarubicin, THP), single chemotherapeutic agent (cisplatin, DDP; gemcitabine, GEM) and combinational treatment (DDP+GEM).

**Figure S3. Establishment of drug sensitivity assay based on tumor spheroids in the microfluidic devices. A** Fluorescence images of negative and positive control. **B** Standard process and viability calculation formula of fluorescence images, S^NC^, negative control, S^PC^, positive control, S^L^ live cells after treatment, S^D^, dead cells after treatment. **C** Representative fluorescence images of spheroids (green, live cells; red, dead cells) treated with different concentrations of cisplatin (DDP) and gemcitabine. Images were taken 24-hour after drug treatment. C0 represent the highest serum concentration of each drug. **D** Spheroids viability measured at the concentration of 10 C0, and this was highly correlated with the IC50 of cisplatin (**E**). BF, bright filed.

**Figure S4 Validation of drug response in patient derived xenografts (PDX).** **A** Correspondence between spheroids, PDX model. **B** Schematic drawing comparing the drug sensitivity assay timeline between PDX model and tumor spheroids in the microfluidic device. **C** Dose response curve of tumor spheroids obtained from patient 19 and 24, spheroids were treated with cisplatin and gemcitabine. **D** The median time of the tumor growth to 10 times the baseline in the control and combination treatment group in PDX-P19and PDX-P24. **E-F** Body weight of mice was slightly decreased in combination treatment groups.

**Figure S5 Detailed information of patients used for spheroids’ drug sensitivity assay.** **A** Baseline characteristics of patients for spheroids’ drug sensitivity assay. Information was collected when the tumor samples were obtained from surgery. **B** Time chart of each patient’s diagnosis and treatment (the starting point was the time of operation at which the sample was collected). TURBT, transurethral resection of bladder tumor; RC, radical cystectomy; RNU, radical nephroureterectomy; GC, gemcitabine and cisplatin or carboplatin; ICIs, immune checkpoint inhibitors.
