# Supplementary figures and images for: Establishment of bladder cancer spheroids and cultured in microfluidic platform for predicting drug response
Source: Bioeng Transl Med. 2023 Dec 4;9(2):e10624. doi: 10.1002/btm2.10624 (PMC10905551; doi:10.1002/btm2.10624)

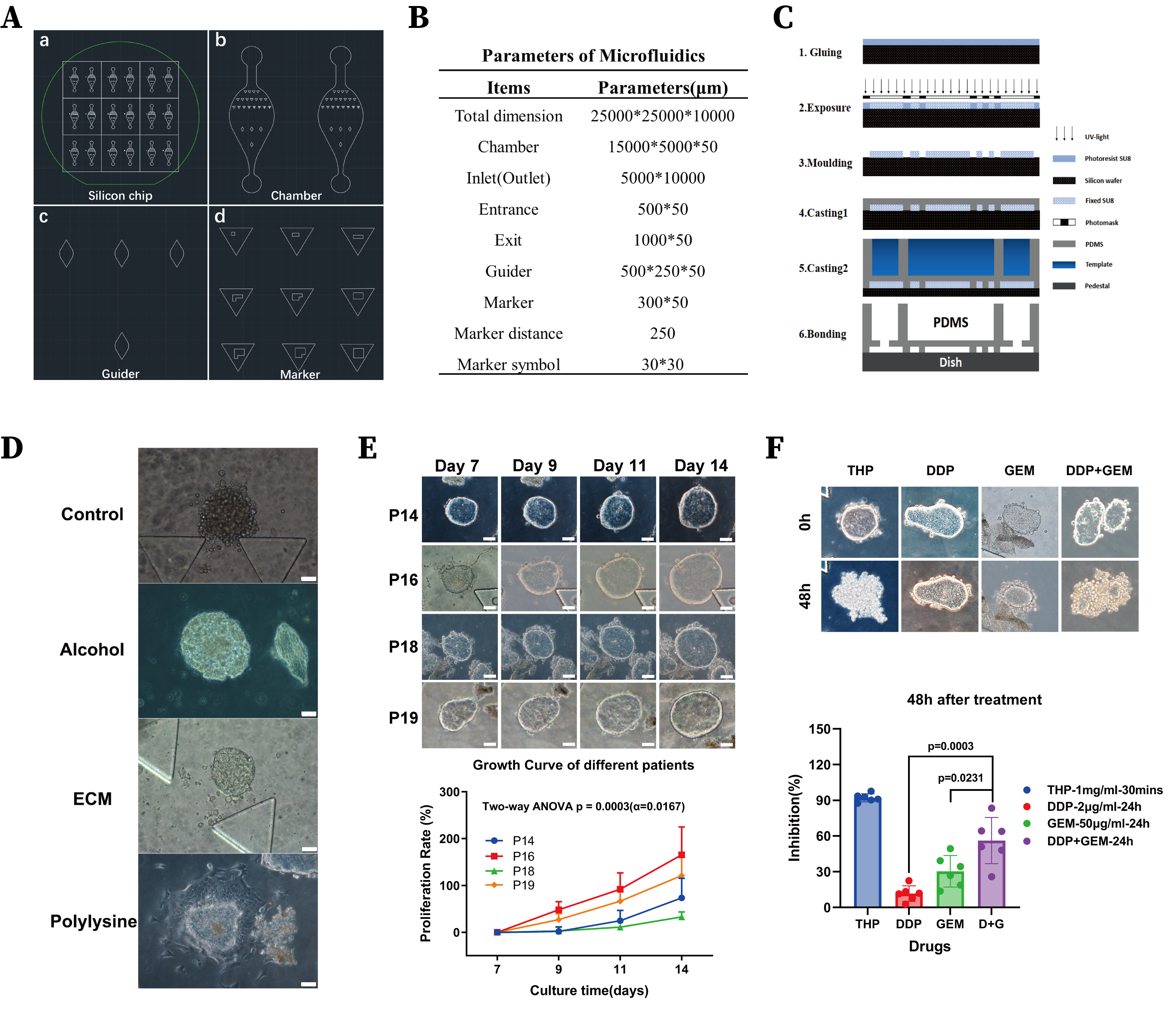

Supplement: Supplementary file 3 — Figure S2: Design microfluidic devices for culture tumor spheroids and drug sensitivity assay. (A) The architecture of the microfluidic device. (B) Parameters of each microfluidic device component. (C) The workflow of constructing a microfluidic device. (D) Effect of different surface modification methods of microfluidic device on spheroids culture. (E) Tumor spheroids obtained from different patients showing distinct growth rate in the microfluidic device. (F) Stimulation of drug sensitivity assay for intravesical instillation (pirarubicin, THP), single chemotherapeutic agent (cisplatin, DDP; gemcitabine, GEM) and combinational treatment (DDP + GEM). [file BTM2-9-e10624-s003.tif]

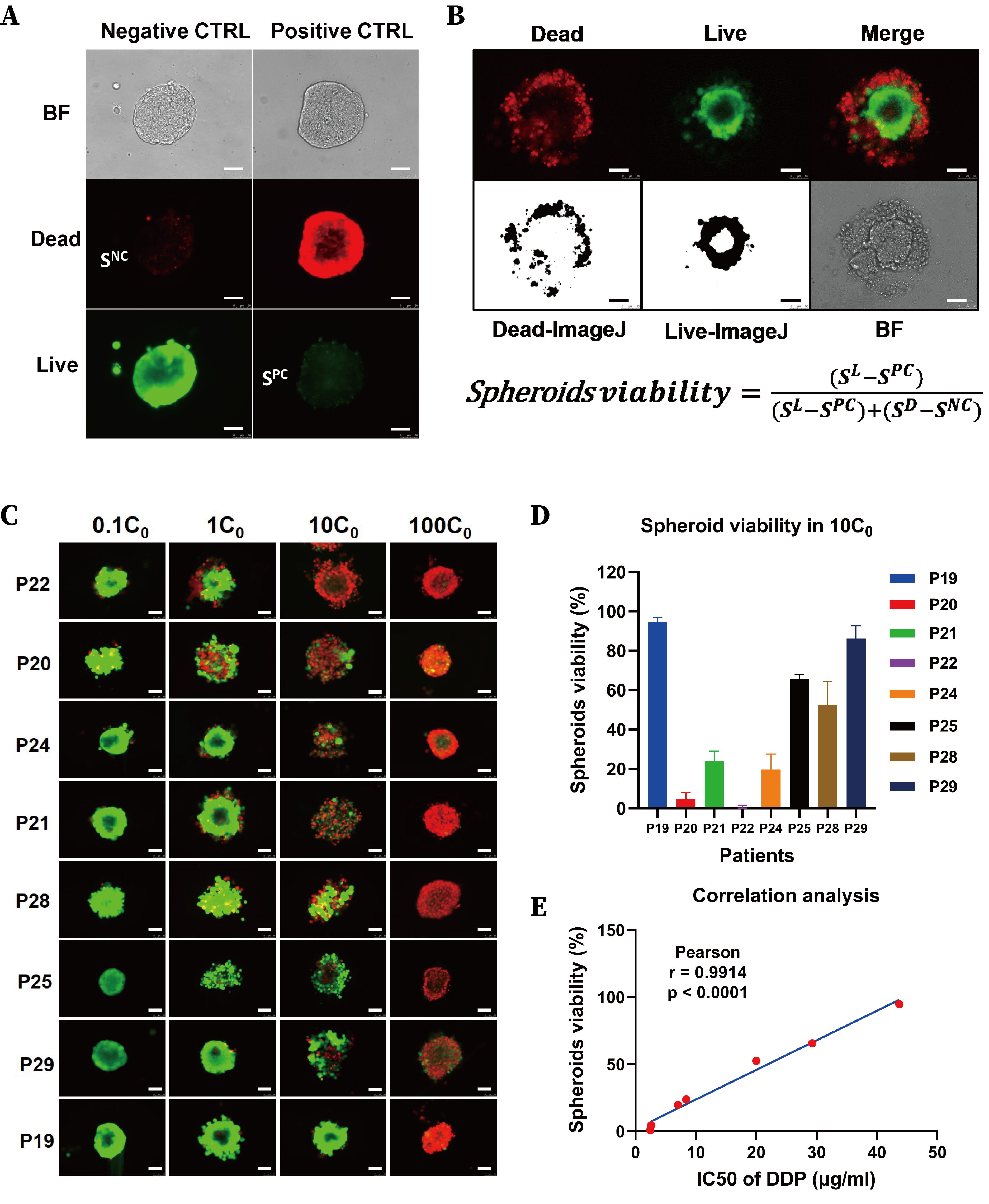

Supplement: Supplementary file 4 — Figure S3: Establishment of drug sensitivity assay based on tumor spheroids in the microfluidic devices. (A) Fluorescence images of negative and positive control. (B) Standard process and viability calculation formula of fluorescence images, SNC, negative control, SPC, positive control, SL live cells after treatment, SD, dead cells after treatment. (C) Representative fluorescence images of spheroids (green, live cells; red, dead cells) treated with different concentrations of cisplatin (DDP) and gemcitabine. Images were taken 24‐hour after drug treatment. C0 represent the highest serum concentration of each drug. (D) Spheroids viability measured at the concentration of 10 C0, and this was highly correlated with the IC50 of cisplatin (E). BF, bright filed. [file BTM2-9-e10624-s006.tif]

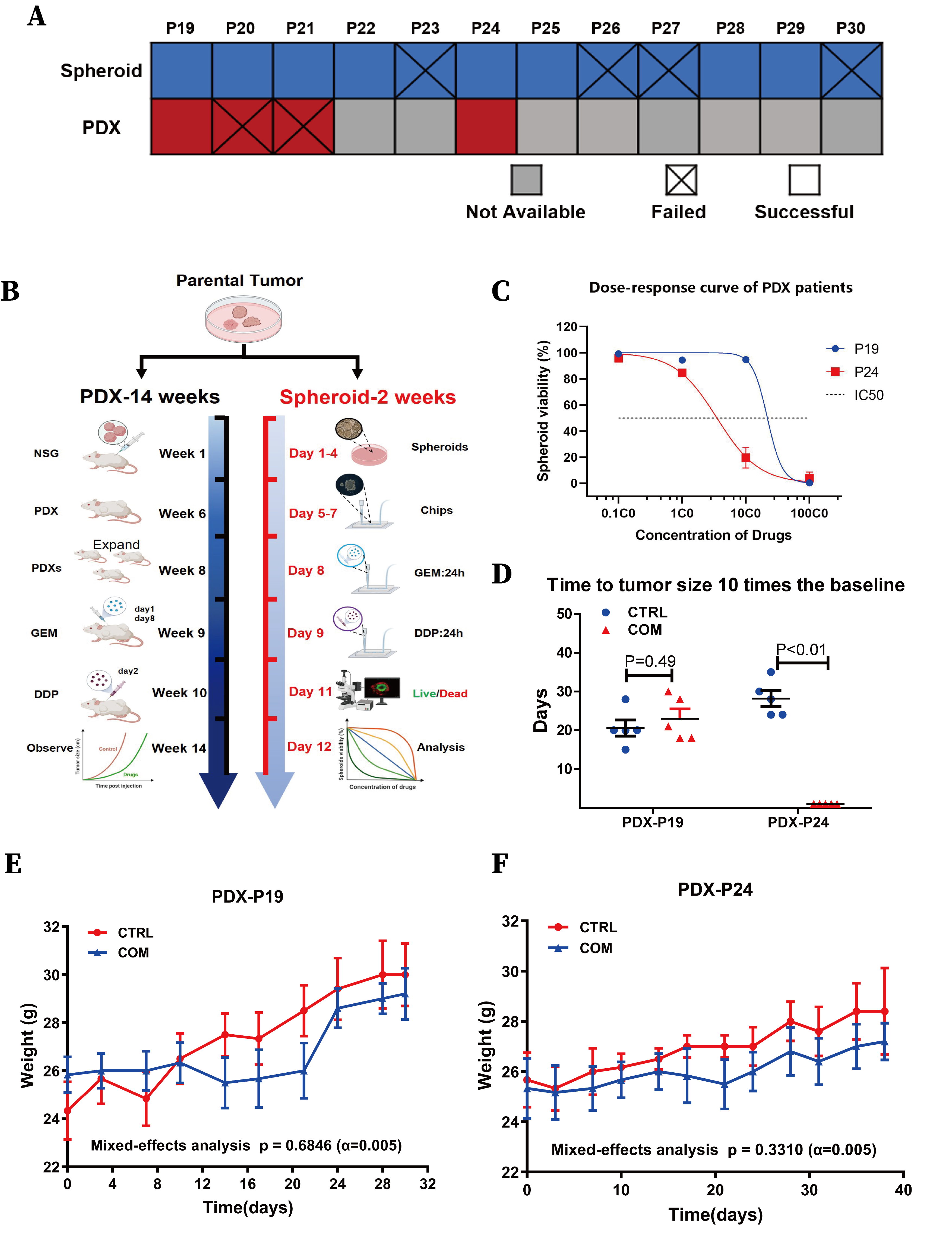

Supplement: Supplementary file 5 — Figure S4: Validation of drug response in patient derived xenografts (PDX). (A) Correspondence between spheroids, PDX model. (B) Schematic drawing comparing the drug sensitivity assay timeline between PDX model and tumor spheroids in the microfluidic device. (C) Dose response curve of tumor spheroids obtained from patient 19 and 24, spheroids were treated with cisplatin and gemcitabine. (D) The median time of the tumor growth to 10 times the baseline in the control and combination treatment group in PDX‐P19and PDX‐P24. (E, F) Body weight of mice was slightly decreased in combination treatment groups. [file BTM2-9-e10624-s001.tif]

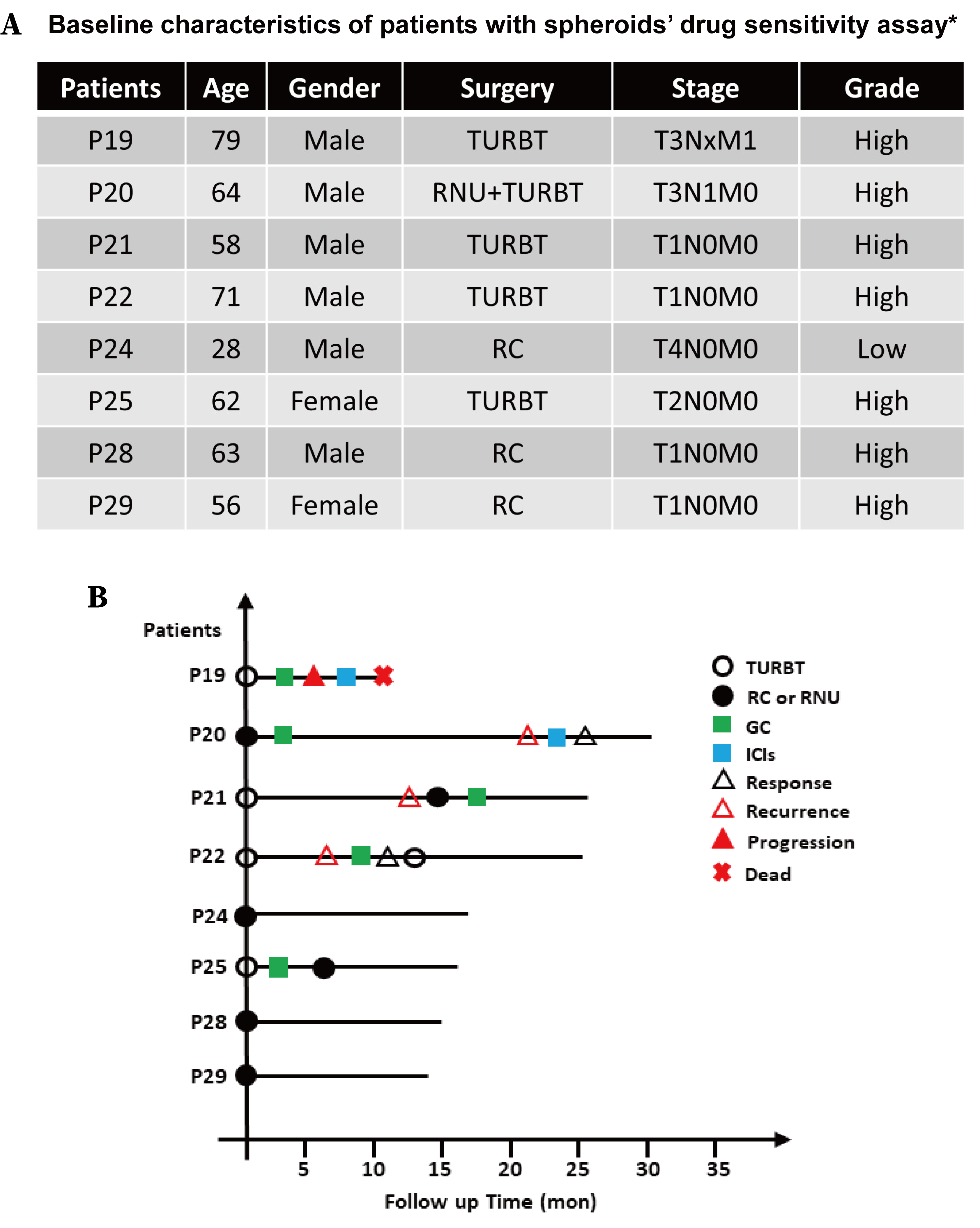

Supplement: Supplementary file 6 — Figure S5: Detailed information of patients used for spheroids' drug sensitivity assay. (A) Baseline characteristics of patients for spheroids' drug sensitivity assay. Information was collected when the tumor samples were obtained from surgery. (B) Time chart of each patient's diagnosis and treatment (the starting point was the time of operation at which the sample was collected). TURBT, transurethral resection of bladder tumor; RC, radical cystectomy; RNU, radical nephroureterectomy; GC, gemcitabine and cisplatin or carboplatin; ICIs, immune checkpoint inhibitors. [file BTM2-9-e10624-s004.tif]
